# Optimization strategies for antimicrobial peptides in dental caries prevention and management: from emerging mechanisms of action to clinical translation

**DOI:** 10.3389/fmicb.2026.1899522

**Published:** 2026-08-14

**Authors:** Hongzhao Ding, Yizinigaer Yasen, Jie Song, Wenjun Fan, Zeyu Wu, Jin Zhao

**Affiliations:** 1 Department of Cariology and Endodontics, The First Affiliated Hospital of Xinjiang Medical University (The Affiliated Stomatology Hospital of Xinjiang Medical University), Urumqi, China; 2 Stomatology Disease Institute of Xinjiang Uyghur Autonomous Region, Xinjiang Medical University, Urumqi, China

**Keywords:** antimicrobial peptides, biofilm disruption, clinical translation, dental caries, peptide optimization

## Abstract

Dental caries remains a prevalent global health burden driven by dysbiosis of the biofilm microbiome. Antimicrobial peptides (AMPs), characterized by a low risk of resistance development and immunomodulatory potential, have emerged as promising alternatives to conventional antimicrobials; however, their clinical translation is still constrained by physiological instability, cytotoxicity, limited oral substantivity, and substantial regulatory and economic barriers. This review systematically summarizes recent advances in the mechanisms of action, rational design, and translational strategies of AMPs for the prevention and management of dental caries. The article first outlines updated mechanistic concepts, highlighting a shift from the classical stable pore-forming model toward transient water channel formation and lipid flip-flop models, and further discusses microbiome-oriented ecological modulation strategies aimed at preserving beneficial commensals and suppressing cariogenic dysbiosis. In addition, structure–activity relationships are examined in depth, with emphasis on how diverse optimization approaches can balance peptide activity, toxicity, stability, selectivity, and manufacturability. The review also delineates the major barriers that impede clinical translation, including production cost, GMP-compatible manufacturing, batch consistency, delivery format, and regulatory definability. Potential strategies are discussed, including molecular farming in plant bioreactors, recombinant or synthetic production platforms, covalent immobilization, *in situ* biomimetic systems, and pH-responsive delivery systems for improving local bioavailability within cariogenic microenvironments. Finally, we emphasize that most current evidence remains preclinical and that no generalizable clinical framework for AMP-based caries prevention has yet been established. Future development should integrate mechanism-guided design, microbiome-level evaluation, standardized safety and efficacy endpoints, scalable manufacturing, and controlled clinical validation.

## 1 Introduction

Dental caries is among the most prevalent chronic oral diseases worldwide. Its development is closely associated with oral microbial dysbiosis, characterized by the enrichment of acidogenic and aciduric communities, including but not limited to *Streptococcus mutans*, and by enhanced community-level acid production, acid tolerance, and biofilm matrix formation (Simón-Soro and Mira, 2015; Zhu et al., 2023; Ye et al., 2025). Conventional anticaries approaches, including chlorhexidine, remain among the most effective antimicrobial agents; however, their broad-spectrum activity may disrupt the commensal microbiota and is associated with adverse effects such as pigmentation of oral tissues (Corbin et al., 2011; Brookes et al., 2020). In contrast, antimicrobial peptides (AMPs), owing to their distinctive mechanisms of action, low propensity for resistance development, and potential functions in remineralization and immunomodulation, are regarded as promising preclinical candidates for next-generation anticaries strategies (Ageitos et al., 2017; Koehbach and Craik, 2019).

However, natural AMPs are readily degraded in the complex oral environment, resulting in poor stability and low bioavailability, which limits clinical application (Drayton et al., 2020). To address these constraints, research has expanded from conventional sequence optimization and structural modification toward function-oriented chemical tailoring, such as phosphorylation to confer remineralization capacity or lipidation to enhance membrane anchoring. In addition, the incorporation of smart drug delivery systems, including liposomes, hydrogels, and nanoparticles, has further enabled targeted release under cariogenic acidic conditions and prolonged mucoadhesion.

Despite continuous technological advances, deeper translational barriers have become increasingly apparent. The high cost of goods sold associated with conventional solid-phase peptide synthesis (SPPS) substantially limits the market penetration of peptide-based oral care products (Chaudhary et al., 2023). In addition, at the level of basic science, recent biophysical studies have challenged the classical transmembrane pore-forming paradigm and have highlighted the central roles of transient water channels and lipid flip-flop in rapid bacterial killing (Koynarev et al., 2025). These findings indicate a need to re-evaluate structure–activity relationships, particularly the interplay between hydrophobicity and antimicrobial potency.

**Figure 1:**
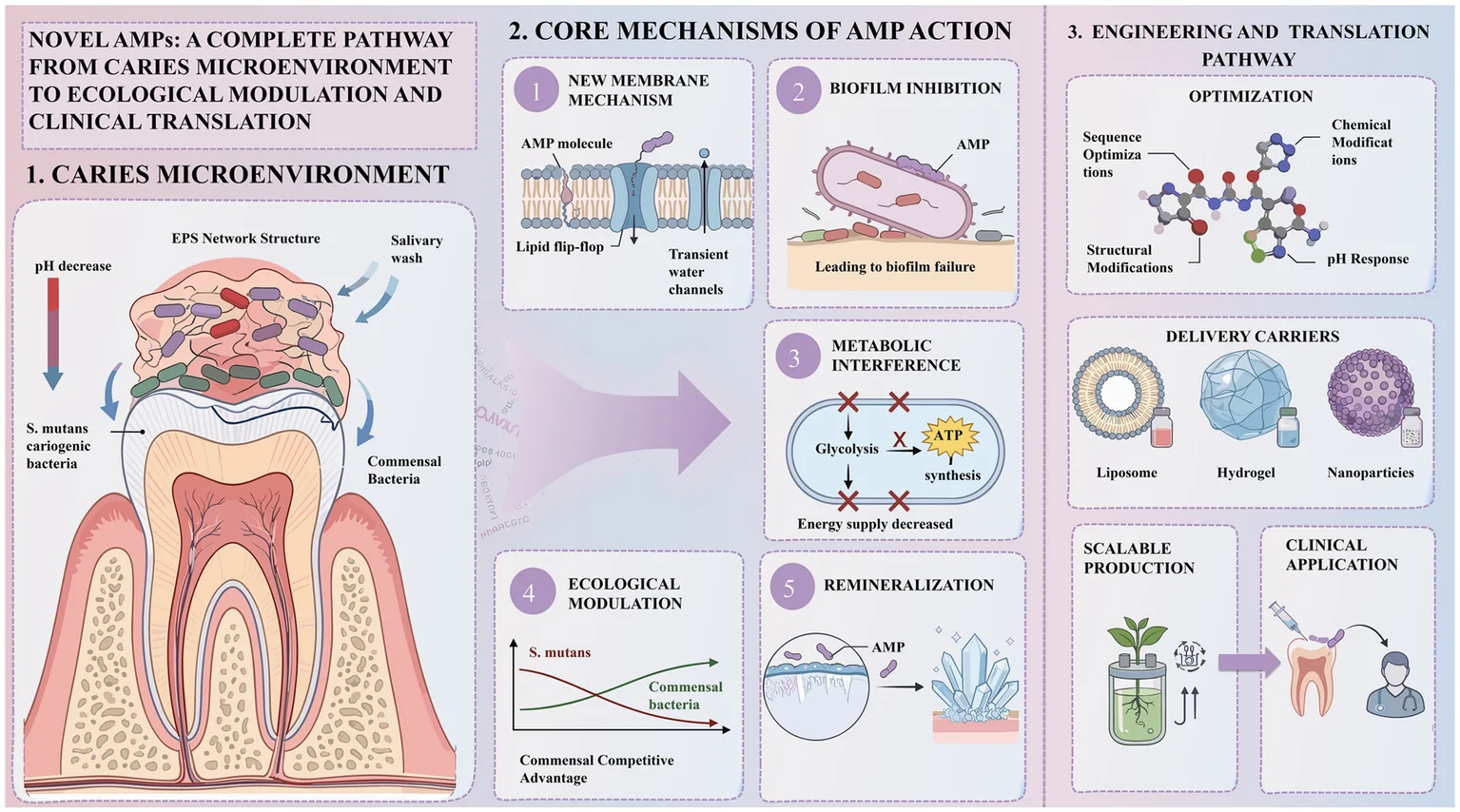
Conceptual roadmap for anticaries AMPs from the cariogenic microenvironment to mechanism-guided engineering and clinical translation.

Building on these considerations, this review systematically summarizes recent advances in the use of AMPs for the prevention and management of dental caries (Figure 1). The discussion first addresses updated concepts, including non-pore membrane perturbation and microbiome-oriented ecological modulation, and then examines how chemical modifications and smart delivery systems can act in concert to overcome limitations in stability and targeting. On this basis, the review further focuses on barriers to clinical translation, evaluating the feasibility of reducing manufacturing costs through plant bioreactors (molecular farming) and mitigating regulatory risks via *in situ* biomimetic strategies (Liu et al., 2016; Daniell et al., 2001; Vetyskova et al., 2025). Finally, the complex relationships among peptide structure, mechanisms of action, anticaries efficacy, toxicity, and stability are critically analyzed to provide a scientific rationale for future clinical translation. Because most available evidence remains preclinical, this review also emphasizes the need for standardized efficacy, safety, microbiome, manufacturing, and regulatory evaluation before AMP-based caries prevention can be generalized to clinical practice.

## 2 Anticaries mechanisms of AMPs

### 2.1 Direct antimicrobial activity

#### 2.1.1 Non-pore membrane disruption

**Figure 2:**
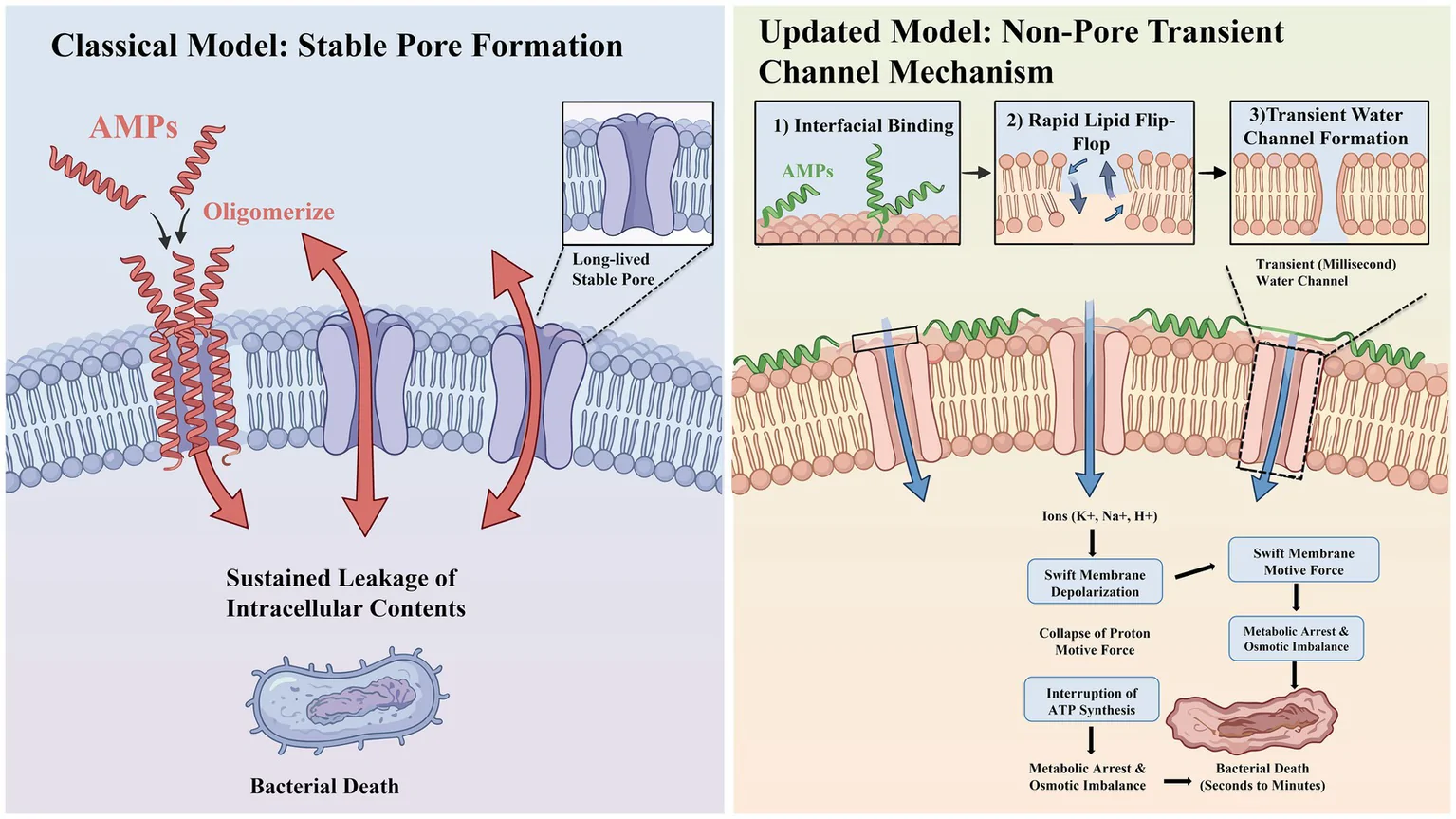
Comparison of classical pore-forming and non-pore transient channel mechanisms of AMPs.

The primary mechanism of AMPs is direct bactericidal activity, which is largely mediated by disruption of the bacterial cell membrane. The traditional view proposes that AMPs must oligomerize on membranes to form stable, protein-like transmembrane pores that induce leakage of intracellular contents (Raheem and Straus, 2019; Luo and Song, 2021; Ganesan et al., 2023; Park et al., 2024). However, this paradigm does not readily explain why many AMPs, including Indolicidin, LL-37, and Aurein 2.2, do not exhibit stable transmembrane structures in experiments yet still trigger extremely rapid ion leakage (Henzler Wildman et al., 2003; Pan et al., 2008; Wimley, 2010; Rokitskaya et al., 2011; Nielsen et al., 2018, 2019). As illustrated in Figure 2, AMPs disrupt bacterial membranes via two mechanisms: the classical pore-forming model, forming stable pores that cause sustained leakage of intracellular contents, and the non-pore transient channel model, which induces rapid lipid flip-flop and millisecond-scale water channels, leading to swift membrane depolarization and metabolic arrest.

To address this discrepancy, Koynarev et al. used time-resolved small-angle X-ray scattering (TR-SAXS) and all-atom molecular dynamics (MD) simulations to identify an alternative mechanism involving lipid flip-flop and transient water channels. Their work indicates that many potent AMPs do not insert deeply into the bilayer. Instead, these peptides preferentially bind to the surface of the outer leaflet. Such binding can catalyze lipid flip-flop, defined as rapid transbilayer movement of lipid molecules, by lowering the associated energy barrier. Lipid flip-flop induces localized membrane perturbations and promotes the formation of transient water channels with very short lifetimes on the millisecond scale. Although these channels are unstable, their frequency is sufficient to permit rapid efflux of ions such as K^+^, Na^+^, and H^+^ along concentration gradients, leading to swift depolarization of the membrane potential. Collapse of membrane potential prevents bacteria from maintaining the proton motive force, interrupts ATP synthesis, and disrupts transmembrane transport, ultimately resulting in metabolic arrest, osmotic imbalance, and cell death (Koynarev et al., 2025). This non-pore model accounts for the observation that certain AMPs can cause membrane depolarization and bacterial killing within seconds to minutes, which is faster than the time typically required for the formation of stable large pores. In the oral cavity, where saliva continuously dilutes and clears antimicrobials, rapid bactericidal activity is particularly important. Accordingly, the design of anticaries AMPs should not rely excessively on increasing hydrophobicity to force deep membrane insertion. A more rational optimization direction is to enhance peptide partitioning at the membrane-water interface and to improve the efficiency of perturbing lipid packing and interfacial membrane organization.

#### 2.1.2 Mechanism-guided design

These advances have also raised a central mechanistic debate: whether non-pore mechanisms can be translated into actionable rules for sequence design. The core concern relates to predictability and reproducibility.

First, the mechanism is highly context dependent. Non-pore processes often exhibit continuum-like behavior. Depending on membrane composition and ionic strength or concentration ratios, a given peptide may shift among interfacial perturbation, transient channel formation, and more pronounced membrane deformation or solubilization. This variability undermines the robustness of deriving fixed sequence rules from a single mechanistic category. Studies showing that short cationic amphipathic peptides induce membrane budding and protrusions under higher loading conditions suggest that some short peptides may converge toward a more general carpet-like disruption pathway in certain settings, further weakening any one-to-one correspondence between mechanism and sequence (Park et al., 2024). Second, there may be orders-of-magnitude differences in the effective peptide-to-lipid ratio between biophysical experiments using model membranes and microbial killing assays. As a result, structural or kinetic conclusions obtained under low-loading conditions may not extrapolate directly to the higher-loading regime required for bactericidal activity in practice. The interfacial activity model was proposed in response to this gap, emphasizing that sequence-to-function mapping should be established under comparable experimental conditions (Wimley, 2010).

Given these divergences, a more cautious interpretation is that non-pore mechanisms can define design objectives with greater clarity, namely enhancing peptide partitioning at the membrane-water interface and accelerating membrane functional destabilization, while remaining insufficient to prescribe a unique sequence. The subsequent discussion of structure–activity relationships will therefore focus on quantifiable parameters linked to interfacial partitioning and membrane perturbation. In particular, it will examine how the hydrophobic moment, updated concepts of hydrophobic matching, and interfacial residues collectively shape antimicrobial efficacy and host selectivity, and will propose sequence optimization strategies that are better suited to the oral environment.

### 2.2 Inhibition of biofilm formation and disruption of established biofilms

Dental caries is a prototypical biofilm-associated disease; therefore, preventing biofilm formation and disrupting established biofilms are key mechanisms underlying the anticaries activity of AMPs. AMPs achieve these effects through multiple pathways. In the inhibition of biofilm initiation, AMPs can reduce initial bacterial adhesion and the production of extracellular polysaccharides (EPS). For example, Temporin-GHa and its derivatives, GHaR and GHa11R, suppress *S. mutans* biofilm formation by decreasing early adhesion and EPS synthesis (Wei et al., 2020). Amyloid fibril peptides are generally considered not to kill bacteria directly. Instead, they self-assemble into gel-like amyloid fibrils that aggregate and sequester bacteria, thereby preventing bacterial attachment to solid surfaces and blocking biofilm assembly and maturation (Chen D. et al., 2021). A peptide derived from the streptococcal adhesins SspA/B, Ssp (A4K-A11K), inhibits *S. mutans* adhesion, potentially by reducing bacterial affinity for saliva-coated polystyrene (Ito et al., 2017). For pre-formed mature biofilms, AMPs can penetrate the biofilm matrix to act directly on bacterial cells or degrade the EPS scaffold (Jiang et al., 2018; Zhou et al., 2021; Luo et al., 2021).

### 2.3 Interference with bacterial metabolism and stress adaptation

In addition to membrane disruption, AMPs may attenuate cariogenicity by modulating bacterial metabolism, acid production, acid tolerance, and stress-adaptation networks. However, these changes require careful interpretation, as reduced enzyme activity or altered gene expression may result from either direct molecular targeting or downstream responses to membrane perturbation and cellular stress. Evidence for direct target binding or enzymatic inhibition generally requires biochemical, structural, genetic, or rescue-based validation, whereas many current studies provide functional or transcriptomic evidence of metabolic suppression (Le et al., 2017).

At the nutrient-acquisition level, one better-characterized example is the membrane-targeted peptide C10-KKWW, which was reported to bind the PtxA component of the phosphotransferase system (PTS), supported by surface plasmon resonance and comparisons among wild-type, ptxA deletion, and complemented *S. mutans* strains (Xiang et al., 2018). Given the role of PTS in carbohydrate uptake and metabolic fitness, these findings suggest that C10-KKWW may interfere with PtxA-associated substrate transport and thereby impair *S. mutans* growth and biofilm viability. Nevertheless, this mechanism represents a specific PtxA-related example and should not be generalized to all anticaries AMPs.

Other studies mainly support functional suppression of acidogenicity and aciduricity. GH12 and the lactotransferrin-derived peptide LF-1 reduced lactic acid production, delayed glycolytic pH drop, decreased LDH and F1F0-ATPase activities, and impaired survival under acidic conditions (Wang et al., 2018; Luo et al., 2023). Similarly, P-113-DPS reduced acid production, acid tolerance, EPS synthesis, and biofilm formation, accompanied by altered expression of virulence-associated genes, including *ldh* and *atpD* (Liu Q. et al., 2025). These findings indicate that AMPs can weaken cariogenic metabolic phenotypes; however, reduced LDH or F1F0-ATPase activity should be regarded as functional inhibition rather than definitive evidence of direct enzymatic targeting.

AMPs may also affect regulatory networks associated with virulence and stress adaptation. GH12, LF-1, and P-113-DPS have been associated with altered expression of genes related to acid production, EPS synthesis, adhesion, quorum sensing, two-component systems, and biofilm formation (Wang et al., 2018; Luo et al., 2023; Liu Q. et al., 2025). More recently, transcriptomic analysis of 8DSS-C8-P-113-treated *S. mutans* suggested broad pathway remodeling involving PTS, ABC transporters, two-component systems, glycolysis, quorum sensing, and other stress-related processes (Zhou et al., 2025). These findings suggest that peptide treatment can reshape bacterial physiology at the pathway level, although such transcriptomic alterations remain associative unless direct peptide–target interactions are experimentally confirmed. Future studies integrating target-binding assays, enzyme kinetics, structural analysis, mutant or rescue experiments, and time-resolved omics will be important for distinguishing direct molecular targets from secondary stress responses.

### 2.4 Enhancement of commensal competitive advantage

According to Marsh’s Ecological Plaque Hypothesis, dental caries fundamentally reflects oral microbial dysbiosis, in which acidogenic and aciduric pathogens progressively displace health-associated commensal communities (Marsh, 1994). Under physiological conditions, commensal oral streptococci such as *Streptococcus sanguinis* and *Streptococcus gordonii* are often ecologically dominant. These species generate H_2_O_2_ via pyruvate oxidase (*SpxB*), thereby imposing oxidative stress on *S. mutans* and weakening its competitive fitness, which contributes to community homeostasis (Cheng et al., 2018; Valdebenito et al., 2018). Physiological levels of H_2_O_2_ can induce oxidative damage through iron-dependent radical formation and can disrupt the glycolytic machinery of *S. mutans* while suppressing the expression of key genes involved in amino-sugar metabolism, including *nagA* and *nagB* (Ye et al., 2025). Collectively, these effects restrict the competitive advantage of *S. mutans* and support microbiome stability. Accordingly, an ideal anticaries strategy should not be limited to broad-spectrum killing but should aim to restore the competitive advantage of commensals while minimizing unnecessary disruption of healthy biofilm architecture.

Within this framework, studies on the GH12 series provide a continuum of evidence spanning mechanistic findings, *in vivo* efficacy, and microbiome-level effects. In a multispecies biofilm composed of *S. mutans*, *S. sanguinis*, and *S. gordonii*, GH12 suppresses *S. mutans* while upregulating *spxB* expression in commensal streptococci and promoting H_2_O_2_ production. This shift strengthens interspecies competitive inhibition and drives the biofilm phenotype toward a less cariogenic state (Jiang et al., 2018). The same work suggests dose-dependent differences in ecological effects, indicating that an optimal concentration window should be defined using ecological readouts. In a rat caries model, a short-duration, high-frequency regimen (5 min, three times daily) with 8 mg/L GH12 reduced lesion severity and decreased the in vivo burden of *S. mutans*. In a paired *in vitro* model using brief, repeated exposure, GH12 concurrently reduced viable bacterial counts, lactic acid levels, and water-insoluble EPS within the biofilm, supporting an anticaries effect associated with sustained suppression of cariogenic phenotypes (Wang Y. et al., 2019; Wang X. et al., 2019). In a human plaque-derived multispecies community that more closely reflects real oral ecology, GH12 inhibited cariogenic traits while exerting relatively limited effects on overall diversity and community structure in plaque from healthy donors, and it showed a partial ecological correction trend under cariogenic conditions (Jiang et al., 2020).

It should be noted that anticaries peptide design inspired by the Ecological Plaque Hypothesis places emphasis on distinct priorities in practice. One strategy focuses on restoring ecological homeostasis by enhancing the competitive advantage of commensals, as exemplified by GH12/LH12 (Jiang et al., 2018; Jiang W. et al., 2023). Another strategy emphasizes narrow-spectrum inhibition of cariogenic pathogens to minimize direct effects on commensals. For instance, specifically targeted AMPs couple a targeting domain to a killing domain to increase selectivity toward *S. mutans* and, with a brief 5 min exposure, can selectively inhibit *S. mutans* biofilms while exerting limited effects on commensal streptococci (Huo et al., 2018). The differences between these two approaches are largely reflected in the primary endpoints and intended scenarios. The former prioritizes maintenance of homeostasis and ecological functional metrics, whereas the latter places greater emphasis on kinetic efficiency under short contact times and selective preservation of commensals. Comparative evaluation of their long-term ecological consequences will require more complex multispecies models and time-resolved study designs.

Recent microbiome studies further support the view that dental caries should be interpreted as polymicrobial dysbiosis rather than a single-pathogen infection. Although *S. mutans* remains an important cariogenic organism, 16S rRNA community analysis has shown that caries-associated communities may also include other taxa, including *S. sobrinus*, *S. salivarius*, *S. parasanguinis*, and *Veillonella*, and that disease progression is often accompanied by reduced community diversity (Gross et al., 2012). Other organisms, such as *Lactobacillus* and *Bifidobacterium*/*Scardovia*, have also been implicated in caries-associated microbial shifts, depending on lesion type, age, diet, and sampling site (Simón-Soro and Mira, 2015; Zhu et al., 2023). In addition, cross-kingdom interactions may contribute to cariogenic biofilm development. For example, *Candida albicans* can coadhere with *S. mutans* on tooth or hydroxyapatite surfaces and may enhance bacterial adhesion and matrix-associated biofilm development, indicating that fungal components should also be considered in selected high-risk or early childhood caries contexts (Metwalli et al., 2013; Zhu et al., 2023).

These findings indicate that the ecological effects of AMPs should be evaluated at both taxonomic and functional levels. In addition to CFU counting or selective reduction of *S. mutans*, future studies should incorporate 16S rRNA sequencing or shotgun metagenomics to assess community composition and functional potential, together with transcriptomic or metabolomic readouts related to carbohydrate transport, glycolysis, acid tolerance, EPS synthesis, alkali generation, and organic acid accumulation. Such multi-omics evaluation would help determine whether AMPs induce durable ecological correction, transient pathogen suppression, or delayed dysbiosis after repeated exposure. For AMP evaluation, these omics-based endpoints would be particularly useful for distinguishing selective ecological modulation from nonspecific community depletion, thereby clarifying whether a peptide restores microbial homeostasis or merely suppresses detectable cariogenic taxa. In this context, pathogen-selective targeting, pH-responsive activation, sub-MIC antivirulence effects, and tooth-surface-restricted exposure may be more consistent with the ecological paradigm of caries management than broadly bactericidal approaches.

### 2.5 Promotion of remineralization and prevention of demineralization

The central pathological feature of dental caries is tooth demineralization. Beyond direct antimicrobial and antibiofilm effects, some anticaries peptides have been designed to integrate antimicrobial activity with remineralization-related functions. It should be noted, however, that not all mineralizing or self-assembling peptides are AMPs in the conventional sense. In this review, peptides with demonstrated antimicrobial or antibiofilm activity are discussed as AMPs or bifunctional antimicrobial–mineralizing peptides, whereas non-antimicrobial self-assembling remineralization peptides are considered adjacent biomimetic peptide systems relevant to caries repair.

Certain AMP-derived peptides can bind hydroxyapatite and serve as nucleation templates for calcium and phosphate ions, thereby facilitating mineral deposition and repairing demineralized lesions. For example, phosphorylated Histatin-5-derived peptides, including Sp-H5 and P-113-DPS, use phosphoserine-containing motifs to enhance tooth-surface binding and calcium recruitment while retaining antibiofilm activity in cariogenic biofilm models (Zhou et al., 2020, 2021). Similarly, GA-KR12 combines the antimicrobial KR12 fragment with gallic acid, enabling bacterial biofilm suppression while promoting mineral deposition along demineralized dentinal collagen (Niu et al., 2021c). More recently, 8DSS-C8-P-113 was designed as a multifunctional AMP containing an antimicrobial domain, an *S. mutans*-targeting domain, and a remineralization domain. This peptide can self-assemble into a tooth-adherent hydrogel, disrupt cariogenic biofilms, resist acid attack, and induce biomimetic remineralization (Zhou et al., 2025). Peptides derived from enamel matrix proteins also provide inspiration for anticaries peptide design. For instance, ameloblastin-derived peptides such as Peptide B and Bm have been reported to exhibit antimicrobial and antibiofilm activity against Gram-positive biofilms (Vetyskova et al., 2025). Because ameloblastin is involved in enamel biomineralization, these findings suggest that mineralization-associated proteins may serve as a source for identifying new AMP candidates. However, current evidence mainly supports the antibiofilm activity of these derived peptides rather than direct remineralization efficacy.

In contrast, P11-4 represents a non-antimicrobial self-assembling remineralization peptide. Kirkham et al. demonstrated that P11-4 undergoes acid- and ion-triggered self-assembly within enamel caries-like lesions and forms a fibrillar scaffold capable of hydroxyapatite nucleation, thereby increasing net mineral gain (Kirkham et al., 2007). Therefore, P11-4 is discussed here not as a conventional AMP, but as an adjacent biomimetic system that informs repair-oriented peptide design. Overall, remineralization-related peptide systems contribute to caries management through distinct mechanisms, including dual antimicrobial–mineralizing activity and purely biomimetic mineral repair.

## 3 Sequence optimizations

For anticaries applications, AMP optimization should be understood as a strategy for matching molecular design to the constraints of local oral use, rather than merely as a means to improve *in vitro* antimicrobial potency. Candidate peptides must remain active in saliva and protease-rich biofilms, tolerate pH and ionic fluctuations, penetrate or persist within extracellular polymeric matrices, avoid excessive cytotoxicity or hemolysis, and minimize unnecessary disruption of health-associated commensals. Different optimization strategies therefore address different translational bottlenecks: charge, hydrophobicity, and amphipathicity tuning mainly refine membrane selectivity and the therapeutic window; truncation, cyclization, and non-proteinogenic amino acid substitution improve manufacturability or proteolytic stability; hybridization, targeting domains, phosphorylation, and functional-group conjugation integrate pathogen selectivity, tooth affinity, and remineralization-related functions; and lipidation, covalent immobilization, and delivery systems improve local retention, exposure control, and biofilm accessibility. In this sense, the following optimization strategies should be interpreted as engineering approaches that connect molecular mechanism with clinical feasibility.

### 3.1 Tuning peptide charge

The antimicrobial activity of AMPs is closely associated with their cationicity and hydrophobicity (Bin Hafeez et al., 2021). Under physiological conditions, AMPs typically carry a net positive charge. Bacterial membranes are enriched in acidic phospholipids such as phosphatidylglycerol, whereas the outer leaflet of mammalian cell membranes is dominated by neutral phospholipids such as phosphatidylcholine, with only a small proportion of negatively charged gangliosides (Matsuzaki, 2009). Increasing positive charge strengthens electrostatic interactions between peptides and negatively charged bacterial surface components, including phosphatidylglycerol and lipopolysaccharide, thereby facilitating membrane disruption (Raheem and Straus, 2019; Satchanska et al., 2024). This electrostatic preference enables AMPs to kill bacteria more readily while exhibiting relatively low cytotoxicity toward mammalian cells, which constitutes the basis of AMP selectivity. Cationicity in AMPs is mainly determined by lysine (side-chain pKa ~ 10.5), arginine (side-chain pKa ~ 12.5), and histidine under mildly acidic conditions (side-chain pKa ~ 6.0). Modulating the number and distribution of these residues provides a practical means to tune peptide cationicity (Gagat et al., 2024). To enhance selective killing against *S. mutans*, histidine residues in Temporin-GHb were replaced with arginine to generate the high-charge-density derivative GHbR. This strategy markedly reinforced electrostatic binding to bacterial membranes, reducing the minimum inhibitory concentration (MIC) and minimum biofilm inhibitory concentration (MBIC) to 1.6 μM and 3.1 μM, respectively (Jiang et al., 2024). Wei et al. designed another derivative, GHaR, by replacing His residues at positions 4 and 11 with arginine, thereby increasing net charge; scanning electron microscopy (SEM) confirmed rapid disruption of bacterial membrane morphology. However, higher charge is not invariably beneficial. For example, further charge enhancement in another derivative such as GHaR9R reduced antimicrobial activity, potentially because disruption of the hydrophobic face or excessive charge clustering impaired effective self-association or membrane insertion. Moreover, although GHaR7R exhibited strong activity, excessive hemolysis limited its suitability as a drug candidate (Wei et al., 2020). This pattern is consistent with the existence of a critical charge density. Above this threshold, peptides may preferentially form transmembrane pores in eukaryotic membranes, increasing hemolysis and overall toxicity (Jiang et al., 2008). Accordingly, structural alterations can shift the antimicrobial mechanism and thereby modulate both activity and toxicity, which often exhibit a trade-off relationship.

Smart peptides can be designed by exploiting pH fluctuations during cariogenesis together with the charge-switching properties of AMPs. The imidazole group in the histidine side chain has a pKa of approximately 6.0. Under physiological oral conditions (pH ~ 7.0), histidine is largely deprotonated, rendering the peptide overall close to neutral; consequently, electrostatic binding to negatively charged bacterial membranes is weak and membrane disruption is limited (Ringstad et al., 2006). In contrast, under cariogenic conditions (pH ~ 5.5), histidine becomes protonated, increasing the net positive charge and promoting strong adsorption to the negatively charged surface of cariogenic bacteria (Gagat et al., 2024). Leveraging this feature, Jiang S. et al. (2023) developed a pH-responsive AMP, LH12, in which histidine residues replace conventional cationic amino acids such as arginine and lysine, thereby enabling selective activation in acidic environments. In an acidic microenvironment dominated by cariogenic bacteria (e.g., pH 5.5), histidine protonation increases the net charge of LH12 from +1.3 to +4.8, markedly strengthening electrostatic interactions with negatively charged bacterial membranes and allowing targeted inhibition of cariogenic species (Jiang W. et al., 2023). GH12 also achieves pH-responsiveness through sequence optimization that introduces multiple histidine residues; through a similar mechanism, this design enhances membrane penetration and disruptive capacity under acidic conditions (Jiang et al., 2021). Translationally, charge tuning is important because it links antimicrobial activation to the cariogenic microenvironment and helps define a usable therapeutic window. Optimized cationicity should therefore be evaluated together with cytocompatibility, hemolysis, and microbiome selectivity, rather than by MIC reduction alone.

### 3.2 Modulating peptide hydrophobicity

#### 3.2.1 Conceptual advances

Hydrophobicity is a central parameter in AMP design. Its spatial distribution, commonly described by the hydrophobic moment, together with size-related effects captured by the concept of hydrophobic matching, jointly determines how peptides interact with bacterial membranes (Hollmann et al., 2016; Zhang et al., 2022; Meier et al., 2023; Conde-Torres et al., 2024). The non-pore mechanism proposed by Koynarev et al. challenges the classical transmembrane pore-forming model, suggesting that bacterial killing by potent AMPs may arise from ion leakage through transient water channels rather than from stable transmembrane pores (Koynarev et al., 2025). This conceptual advance necessitates a reevaluation of the roles of hydrophobic moment and hydrophobic matching in AMP design.

#### 3.2.2 Hydrophobic moment and interfacial activity

The hydrophobic moment represents the vector sum of amphipathicity along a peptide chain and is a key determinant of interfacial activity under non-pore mechanisms (Eisenberg et al., 1982; Reißer et al., 2014). Unlike the traditional view in which the hydrophobic moment primarily facilitates oligomerization into pores, emerging evidence suggests that a high hydrophobic moment mainly drives AMPs to adopt a parallel orientation on the surface of the outer leaflet. This pronounced amphipathic segregation not only enables peptides to recognize and bind bacterial membranes with specific curvature but may also lower the energy barrier for lipid flip-flop through interfacial binding. For example, Zhang et al. optimized the topological arrangement of the H-R peptide series using the helical periodicity of 3.6 residues per turn. The resulting increase in hydrophobic moment enhanced curvature sensing on bacterial membranes, maintaining high antimicrobial activity while conferring favorable selectivity toward host cells (Zhang et al., 2022). Studies on ZXR-2 further showed that increasing the hydrophobic moment through amino acid substitutions can markedly strengthen selective binding to bacterial membranes, converting the peptide from a pro-apoptotic antitumor agent into a potent anticaries AMP (Zhou et al., 2016; Chen et al., 2017). Collectively, these findings indicate that topological optimization to increase the hydrophobic moment can improve adsorption stability and perturbation efficiency at the membrane interface.

#### 3.2.3 Hydrophobic matching and tryptophan anchoring

The concept of hydrophobic matching has also evolved from strict geometric length matching to compatibility with the interfacial chemical environment. Classical theory posits that the length of the peptide hydrophobic segment must closely match the thickness of the membrane hydrophobic core to maintain pore stability; otherwise, membrane deformation or peptide aggregation may occur (Grau-Campistany et al., 2015; Gagnon et al., 2017; Meier et al., 2023). However, given that potent AMPs primarily act at the membrane interface and induce transient channels with millisecond lifetimes, strict geometric correspondence between peptide length and bilayer thickness is no longer a prerequisite. Design priorities should instead shift toward the compatibility of hydrophobic residues with the chemical environment at the membrane interface. For example, tryptophan exhibits a distinctive interfacial preference due to its indole ring, which can be positioned at the lipid-water interface through *π*-cation interactions rather than penetrating deeply into the hydrophobic core (Yau et al., 1998; Norman and Nymeyer, 2006). In the design of [W7]KR12-KAEK, Da Silva et al. (2017) replaced isoleucine at position 7 with tryptophan, leveraging this interfacial anchoring property to markedly enhance penetration and clearance of *S. mutans* biofilms. In addition, Wei et al. (2020) substituted leucine with tryptophan in the Temporin-GHa derivative GHaR9W, which not only strengthened targeted insertion through the indole ring but also avoided structural destabilization or nonspecific hemolysis associated with excessive hydrophobic core reinforcement, as observed for GHaR9R.

Accordingly, hydrophobic modulation strategies for anticaries AMPs are shifting from promoting deep insertion and transmembrane pore formation toward optimizing interfacial binding and membrane potential perturbation. Future molecular design should prioritize increasing the hydrophobic moment through precise amino acid patterning and, following the approaches used for [W7]KR12-KAEK and GHaR9W, incorporate interfacial residues such as tryptophan to refine peptide positioning at the membrane surface. This strategy can impair the metabolic function of cariogenic bacteria by inducing lipid flip-flop and transient water channels without requiring stable transmembrane pores, thereby preserving antimicrobial efficacy while reducing the risk of toxicity to host cells.

### 3.3 Amphipathicity-tuning strategies

Some AMPs are amphipathic, with hydrophilic, charged regions and hydrophobic regions segregated within the same peptide chain or conformation. This spatial separation enables preferential binding to negatively charged bacterial membranes, followed by disruption of membrane integrity (Bui Thi Phuong et al., 2024). More pronounced amphipathicity is often associated with stronger antimicrobial activity, but cytotoxicity, including hemolysis, may also increase, necessitating fine coordination among charge, hydrophobicity, and amphipathic patterning (Edwards et al., 2016). This coordination is not a simple additive effect of bulk properties but is achieved through precise substitutions at specific residues to remodel charge distribution and the hydrophobic moment at a microscopic level. For example, during optimization of Temporin-GHa, alanine at position 8 was replaced with arginine to generate the derivative GHaR8R. This modification increased the net positive charge to +3 and reduced hydrophobicity (GRAVY) from 1.115 to 0.631. This calibrated balance between increased cationicity and decreased hydrophobicity strengthened electrostatic targeting of negatively charged bacterial membranes while reducing permeabilization of neutral erythrocyte membranes, resulting in high selective antimicrobial activity (cell selectivity index, CSI = 27) (Wei et al., 2020). Zhang et al. designed three AMPs, GH8, GH12, and GH16, based on the sequence template (XXYY)_n_, in which peptide length was adjusted to tune the charge-hydrophobicity balance. Among them, GH12 (GLLWHLLHHLLH-NH2) carries a net charge of +4 and has a hydrophobic content of 58.3%, and it showed the most potent inhibition of cariogenic bacteria, with a MIC of 4–8 μg/mL, consistent with an optimized charge-to-hydrophobicity ratio (Wang et al., 2017). In this design, Trp was fixed at position 4 at the hydrophilic-hydrophobic interface, ensuring membrane anchoring and membrane disruption even as overall charge and hydrophobicity varied.

This residue-level control logic has been extended to spatial scale optimization. To maximize amphipathicity without increasing toxicity, the design of H-R peptides (e.g., RRWFRRWRRWFR) moved beyond the conventional (XXYY)_n_ repeat pattern and instead applied topological optimization based on the helical periodicity of 3.6 residues per turn. This phase alignment places cationic and hydrophobic residues on opposite faces of the helix, generating an exceptionally high hydrophobic moment (Zhang et al., 2025). Such precision not only governs bactericidal efficiency but also appears to involve threshold effects that can shift the dominant mechanism of action.

In complex pathological settings such as caries, amphipathicity modulation has evolved toward more adaptive designs. To address the negatively charged extracellular polysaccharide (EPS) matrix, increasing the proportion of lysine can raise charge density and promote penetration of the viscous matrix through strong electrostatic driving forces. This can be combined with short hydrophobic motifs, such as Trp-Trp branches, to enable physical disruption of bacteria embedded within mature biofilms (Jiang et al., 2024). In the caries field, these amphipathicity-based strategies have also been extended from bioactivity to surface modification. For example, peptides have been engineered with the cationic terminus electrostatically anchored to dentinal collagen while the hydrophobic terminus is directionally arranged to form a physical water-repellent barrier, thereby achieving antimicrobial protection while mitigating clinical problems related to moisture-induced interface degradation and bacterial breakdown at restorative sites (Moussa et al., 2019a, 2019b). Thus, amphipathicity tuning provides a direct bridge between antimicrobial selectivity and material-interface performance. For clinical translation, the key objective is not maximal amphipathicity, but a reproducible balance among bacterial membrane disruption, host-cell compatibility, and durability at tooth-restoration interfaces.

## 4 Structural modifications

### 4.1 Length optimization

#### 4.1.1 Reframing the length effect

Peptide length has long been regarded as a key determinant of AMP mechanism of action (Dong et al., 2012; Wang et al., 2017; Wang, 2020). In classical pore-forming models, *α*-helical AMPs are proposed to require a minimum length threshold, typically >22 amino acids (Chen and Jiang, 2023). This requirement is based on geometric considerations: the hydrophobic core of the bacterial membrane is approximately 30 Å thick, whereas a canonical α-helix rises by about 1.5 Å per residue, implying that roughly 20 residues are needed to span the bilayer and form a stable transmembrane pore (Grau-Campistany et al., 2015). However, this framework does not readily explain the potent bactericidal activity of short peptides such as Indolicidin (Rokitskaya et al., 2011). Recent non-pore mechanisms suggest that peptide length design need not be constrained by strict geometric matching to membrane thickness. Instead, design should emphasize optimization of the efficiency of interfacial perturbation. Short peptides, with higher diffusion rates and strong interfacial activity, can rapidly induce localized curvature changes and disrupt transmembrane ion gradients, thereby enabling rapid killing (Li et al., 2017; Park et al., 2022; Schmidt and Wong, 2013). Accordingly, length optimization should shift from simple size extension toward precise tuning of interfacial activity and structural stability.

#### 4.1.2 Peptide truncation

Based on non-pore mechanisms, shortening peptide length is an effective approach to enhance interfacial activity and reduce translational barriers. Although short peptides are unlikely to form stable transmembrane structures, their high charge density and amphipathic organization can be sufficient to promote transient water channel formation at the membrane interface, leading to rapid depolarization of bacterial membrane potential. For example, the 13-residue peptide Indolicidin exhibits an ion leakage rate induced by interfacial binding that is comparable to that of longer peptides (Grau-Campistany et al., 2015). From a sequence design perspective, truncation can markedly reduce synthesis costs and immunogenicity, and it may decrease hemolytic toxicity by limiting nonspecific hydrophobic insertion (Chen S. P. et al., 2021). Chen et al. (2019) truncated the 37-residue LL-37 to generate the 13-residue peptide IG-13-1. While preserving the core bactericidal motif, this short peptide displayed faster killing kinetics than the parent peptide and completely eradicated *S. mutans* within 15 min. Similarly, Ahn et al. synthesized HBD3-C15 from the 15 C-terminal residues of HBD3, demonstrating that this fragment retained substantial antibiofilm activity and acted synergistically with chlorhexidine (Ahn et al., 2017). In contrast, extending GH12 to GH16 did not improve anticaries activity; GH16 showed markedly weaker activity against *S. mutans*, indicating that peptide length optimization requires an appropriate hydrophobic–cationic balance rather than simple sequence extension (Wang et al., 2017). These studies indicate that retaining core sequence motifs with high interfacial activity can be sufficient to achieve potent antimicrobial effects through perturbation of membrane permeability. From a translational standpoint, truncation is particularly attractive when it preserves antibiofilm activity while reducing peptide length, synthesis cost, and quality-control complexity.

#### 4.1.3 Peptide elongation

Although transmembrane spanning is no longer considered a prerequisite, peptide elongation remains important for enhancing secondary-structure stability and enabling multifunctionality. Under non-pore mechanisms, longer sequences may not penetrate deeply into the bilayer, yet they can facilitate the formation of a stable amphipathic *α*-helix. This conformation provides a larger membrane-binding interface and may more effectively lower the energy barrier for lipid flip-flop. Elongation is therefore often used to build functional domains or to improve adaptability under specific conditions. For example, the synthetic peptide [W7]KR12-KAEK was extended by four residues at the C terminus. This modification was not intended to promote pore formation but to optimize the balance between hydrophobic and hydrophilic moments, thereby improving penetration through extracellular polysaccharide matrices and increasing anchoring efficiency at the membrane surface (Da Silva et al., 2017). Similarly, adding an N-terminal helix-capping motif (GKLI) to the lactoferrin-derived peptide LF-2 increased peptide length, but the primary effect was stabilization of the helical conformation, which enhanced proteolytic stability and bioactivity at the membrane interface (Luo et al., 2021). In addition, longer peptides can provide steric effects and multivalent binding capacity, which supports multifunctional designs. For instance, histatin-derived peptides have been reported to combine antimicrobial activity with the ability to induce hydroxyapatite remineralization (Niu et al., 2021b). Accordingly, peptide elongation should be guided by integration of functional modules and improvement of conformational stability.

### 4.2 Cyclization strategies

In the complex oral microecological environment, proteases present in saliva and secreted by bacteria are major drivers of rapid degradation and loss of activity of native linear AMPs. Accordingly, introducing conformational constraints, particularly cyclization, is considered a central strategy to improve *in vivo* stability. Cyclization restricts the conformational freedom of the peptide backbone through the introduction of covalent linkages, thereby reducing exposure of protease cleavage sites while optimizing bioactivity. Depending on the bonding chemistry, cyclization approaches include head-to-tail cyclization, disulfide-mediated cyclization, cyclic dipeptides, and side-chain cyclization (Zhang et al., 2023c). Daniell et al. expressed the cyclic scaffold peptide retrocyclin (RC101) using a chloroplast expression system and showed that the cyclic architecture markedly increased resistance to proteolytic hydrolysis while maintaining strong activity against *S. mutans* and its biofilms under oral conditions (Liu et al., 2016). To further enhance antimicrobial performance, Zhang et al. (2016) proposed a combined cyclization-lipidation strategy. The resulting cyclic lipopeptides used cyclization to lock conformational stability and incorporated fatty acid chains to strengthen hydrophobic interactions, achieving robust clearance of cariogenic biofilms and downregulation of associated virulence genes such as gtf (Min et al., 2017).

However, excessive conformational rigidity may compromise antimicrobial activity, and this stability–activity trade-off has attracted increasing attention. Koehbach et al. compared a linear peptide with its grafted cyclotide variants and found that, although grafting extended the serum half-life by more than 30-fold, the killing kinetics against multidrug-resistant bacteria were markedly slower than those of the original linear peptide (Koehbach et al., 2021). This observation suggests that an overly rigid scaffold may introduce steric constraints that hinder productive engagement between the active motif and the bacterial membrane. Consistent with this view, Ding et al. reported a reverse-optimization example in which the native cyclic peptide bactenecin was linearized and truncated to generate Bac8c. This derivative showed stronger anticaries activity and biofilm inhibition than the cyclic parent peptide, with lower cytotoxicity (Ding et al., 2014). Although cyclization is an effective approach to mitigate proteolytic degradation in the oral cavity, anticaries peptide design should not pursue structural stability indiscriminately. Future optimization should aim to identify an optimal balance between cyclic rigidity for protease resistance and the conformational flexibility required for membrane disruption, or select cyclization versus linearization in a target-informed manner. This balance is central to translation because improved protease resistance is clinically meaningful only when it is accompanied by retained killing kinetics, scalable synthesis, and consistent structural characterization.

### 4.3 Hybrid peptides

Hybridization modifies peptides by fusing distinct functional domains through linkers, aiming to overcome the limited selectivity and single-function profiles of native AMPs in the complex oral environment. This strategy has been used primarily to address targeting. Eckert et al. developed the targeted hybrid peptide C16G2 by incorporating a fragment derived from an interspecies signaling molecule of *S. mutans* (CSPC16). In mixed-species communities, C16G2 showed high discriminatory capacity, selectively eradicating *S. mutans* without reducing total bacterial load or the abundance of beneficial streptococci such as *Streptococcus gordonii*. These findings support the concept that removal of a key pathogen can drive microbiome remodeling from a diseased state toward a healthier configuration (Guo et al., 2015). Nevertheless, targeted killing alone is insufficient to fully restore damaged hard tissues. Consequently, the development of mineralizing hybrid peptides that integrate biofilm control with enamel repair has become a major research focus.

Unlike targeted hybrid peptides, mineralizing hybrid peptides face a central design dilemma: the inherent functional antagonism between a cationic antimicrobial domain (AMP domain) and an anionic mineralization domain (Min domain) (Huang and Horng, 2019; Lunkad et al., 2021). Antimicrobial activity typically depends on a high density of positive charges to enable electrostatic capture of bacterial membranes, whereas mineralization relies on negatively charged groups to chelate Ca^2+^. In conventional designs exemplified by TVH19, rigid linkers are mainly used to physically separate the two domains, thereby avoiding conformational collapse caused by intramolecular attraction between opposite charges (Han et al., 2021). In contrast to this avoidance strategy, Zhou et al. developed 8DSS-C8-P-113 and proposed a rational scheme in which charge antagonism is harnessed to drive assembly. Rather than suppressing electrostatic interactions, this design introduced a hydrophobic core (C8) and precisely tuned the isoelectric point, converting electrostatic attraction, which would otherwise lead to inactivation, into a driving force for supramolecular self-assembly. Within such supramolecular architectures, the opposing charges are spatially re-distributed through ordered intermolecular packing, thereby mitigating intramolecular antagonism and preserving function (Zhou et al., 2025). Hybrid and targeting-domain designs are therefore especially relevant to caries translation because they move AMP development from broad-spectrum killing toward disease-site-oriented intervention. By combining pathogen selectivity, biofilm control, tooth affinity, and remineralization-related functions, these systems more closely match the multifactorial requirements of caries management.

## 5 Chemical modifications

### 5.1 Non-proteinogenic amino acid substitution

Native AMPs are composed of L-amino acids and are susceptible to degradation by oral proteases, including salivary enzymes. Replacing L-amino acids with D-amino acids can enhance proteolytic stability and improve resistance to enzymatic degradation (Jia et al., 2017; Lu et al., 2020). D-amino-acid modification is mainly implemented as full-sequence D-enantiomerization or as site-specific D-residue substitution. D-GL13K is the D-enantiomer of L-GL13K. Substitution of L- with D-amino acids markedly improves resistance to proteolysis, including degradation by oral trypsin, while preserving antimicrobial activity (MIC = 1 μg/mL) (Moussa et al., 2019b). Hu et al. (2024) designed DJK-5@SVA, in which the antimicrobial domain DJK-5 was constructed with D-amino acids to enhance protease resistance while retaining intrinsic antimicrobial activity. Overall, full-sequence D-enantiomerization tends to provide robust stability gains in anticaries applications.

Site-specific D-residue substitution can also substantially improve stability. For example, CLP-4 contains four non-native D-amino acids. Incorporation of these residues reduces susceptibility to cleavage at protease-sensitive sites, thereby enhancing resistance to enzymes such as trypsin and chymotrypsin and improving molecular stability (Min et al., 2017). Despite these advantages, D-amino-acid substitution also presents challenges. For membrane-active AMPs, partial D-residue substitution may reduce antimicrobial activity and, in some cases, increase toxicity (Lu et al., 2020). This may occur because introducing D-residues can disrupt the native conformation of the peptide, with outcomes depending on the number and positions of substitutions. In contrast, full-sequence D-enantiomerization generally preserves membrane-disruptive mechanisms (Gan et al., 2021). These observations indicate that structural modifications can directly influence peptide stability in the oral cavity, and antimicrobial potency is affected indirectly through structure-dependent shifts in mechanism. The use of other non-proteinogenic amino acids in anticaries AMPs remains underexplored. For example, Aib (*α*-aminoisobutyric acid) and 2-naphthylalanine (2-Nal) can increase hydrophobicity and conformational stability. Such modifications may enhance biofilm penetration and prolong activity in saliva, although systematic evidence in the context of caries prevention and treatment is still limited (Lu et al., 2020; Oliva et al., 2018).

### 5.2 Phosphorylation modification

Phosphorylation is a key post-translational modification that alters the conformation and functional properties of biomacromolecules through covalent attachment of phosphate groups, typically at the hydroxyl sites of serine, threonine, and tyrosine residues (Newcombe et al., 2022; Zhong et al., 2023). Phosphorylation can substantially improve the stability of AMPs while reducing hemolytic toxicity (Ba et al., 2024). Notably, in oral biomedical research, serine-specific phosphorylation has shown distinctive translational value. Phosphorylated peptides can bind tooth hydroxyapatite crystals via phosphate groups, thereby enhancing targeted adhesion to dental tissues and promoting oriented hydroxyapatite deposition. This provides a mechanistic basis for remineralization-oriented therapies. Early attempts, such as DPS-PI, introduced diphosphoserine (DPS) to markedly increase peptide affinity for hydroxyapatite (HA), primarily through strong electrostatic interactions between phosphate anions and calcium ions on the tooth surface (Zhang et al., 2019).

However, adsorption alone is insufficient to repair demineralized tissues. Subsequent studies advanced from passive binding to active repair. Sp-H5 and P-113-DPS, developed by Zhou and Niu et al., use phosphorylated moieties not only as anchoring sites but also to create an ion-capturing layer on enamel surfaces. By attracting salivary calcium ions, these peptides form nucleation templates that successfully induce *in situ* enamel remineralization (Zhou et al., 2020, 2021). Importantly, P-113-DPS not only binds dental tissues but also modulates gene expression in *S. mutans*, indicating that phosphorylation can confer remineralization capability without compromising targeted suppression of bacterial virulence (Liu Q. et al., 2025). This modification strategy clarifies a design concept for bifunctional AMPs: a cationic peptide segment disrupts bacterial biofilms, whereas an anionic modification motif helps resist acid erosion and promotes tissue repair.

### 5.3 Small-molecule and functional-group conjugation

#### 5.3.1 Multifunctional applications of gallic acid

Conjugation of pharmacologically active natural small molecules to AMPs has emerged as a promising approach to improve peptide stability and broaden functionality. The underlying rationale is to leverage the distinctive chemical properties of small molecules to compensate for intrinsic limitations of peptides. On the one hand, small molecules can enhance stability. Fu et al. modified the N terminus of the targeted peptide C16G2 with gallic acid (GA-C16G2). Incorporation of gallic acid effectively masked aminopeptidase-sensitive degradation sites, markedly prolonging the peptide half-life in saliva, while the inherent membrane-perturbing property of gallic acid also contributed to enhanced bactericidal activity (Fu et al., 2025). On the other hand, small molecules can confer new mineralization functions. Zhang et al. developed GAPI by exploiting the metal-chelating capacity of the pyrogallol moiety in gallic acid (Zhang et al., 2023b). The study showed that GAPI achieved >94% retention in human saliva after 60 min through N-terminal modification. More importantly, the conjugated group formed coordination complexes with Ca^2+^, serving as crystal nucleation sites and significantly improving the crystallinity and Ca/P ratio of the remineralized enamel layer under pH cycling conditions. In addition, the scope of small-molecule conjugation has expanded from enamel remineralization to the more complex challenge of deep dentin repair. Because dentinal caries management requires not only mineral deposition but also preservation and restoration of the organic collagen scaffold, Niu et al. developed the bifunctional peptide GA-KR12 (Niu et al., 2021c). In this design, gallic acid (GA) was grafted to the N terminus of the human-derived antimicrobial fragment KR12, yielding a molecule with combined collagen-protective and mineralization-inducing properties. Experimental evidence indicated that GA-KR12 effectively disrupts the cell membrane of *S. mutans* and suppresses biofilm growth. More importantly, the pyrogallol moiety of GA functions as a Ca^2+^ capture motif, enabling GA-KR12 to induce functional remineralization on demineralized dentinal collagen fibrils. Fourier transform infrared spectroscopy (FTIR) and micro-computed tomography analyses further showed that GA-KR12 markedly reduced collagen degradation, reflected by decreased loss of the amide I signal, and successfully guided ordered deposition of hydroxyapatite crystals along the collagen scaffold.

Mineralization-oriented modifications, such as phosphorylation or conjugation with pyrogallol-containing motifs, can enhance HA binding and ion-capturing capacity, but they may also introduce time-dependent changes in functional allocation. As the molecule continuously enriches Ca/P and participates in nucleation and deposition, the fraction in a freely diffusible state may decrease, whereas the surface-bound fraction may increase. During progressive thickening of the mineralized layer, partial embedding or shielding may occur. Under these conditions, mineralized AMPs may experience an effective concentration drop in the later phase, potentially weakening the rapid, contact-dependent antimicrobial action (Yasir et al., 2020; Tian et al., 2025). This potential trade-off can be analogized from an interfacial accessibility perspective. The antibiofilm peptide G3 relies on rapid binding to bacterial surfaces during the adhesion-inhibition stage, whereas during mature biofilm clearance it co-localizes and interacts with the eDNA scaffold, indicating that accessibility of the target interface is itself a critical determinant of antibiofilm performance (Zhang et al., 2020). Meanwhile, as mineral layers form and thicken, the accessibility of antimicrobial domains at the interface may decrease, and membrane-perturbing motifs may become progressively limited by steric shielding or conformational constraint (Bagheri et al., 2009).

Accordingly, for mineralization-conjugation systems, time-resolved antimicrobial kinetics and measurements of interfacial accessibility are required to incorporate target accessibility into parameterized evaluation. It should be emphasized that the time-dependent trade-off and reduced accessibility described above remain testable mechanistic hypotheses. Current studies predominantly report endpoint antimicrobial and endpoint remineralization outcomes, whereas systematic quantification of how antimicrobial kinetics evolve during the mineralization process remains limited (Zhou et al., 2021; Zhang et al., 2023a). A more conservative conclusion is therefore that bifunctional systems may exhibit partial overlap and interference between antimicrobial and mineralization phases, and future work should validate and parameterize these dynamics using time-resolved functional assays to support more predictable molecular design.

#### 5.3.2 Hydrophobic side-chain functionalization

In contrast to gallic acid, which confers mineralization capacity through its polyhydroxyl structure, another class of small-molecule conjugation strategies focuses on introducing hydrophobic aromatic groups to alter peptide surface properties and recognition behavior. Simon et al. used cyclic dipeptides as scaffolds to investigate how side-chain chemical modifications affect bioactivity. They found that the unmodified native peptide lacked inhibitory activity against *S. mutans*, whereas chemical conjugation of a hydrophobic benzyl group to the tyrosine side chain immediately conferred pronounced antibiofilm activity. Removal of the benzyl modification abolished activity, indicating that this exogenous aromatic fragment is a decisive determinant of the peptide’s specific targeting capability (Simon et al., 2019). This strategy does not primarily rely on intrinsic membrane-lytic toxicity of the peptide backbone. Instead, the benzyl group is proposed to introduce steric and hydrophobic effects that selectively interfere with bacterial quorum-sensing pathways, thereby suppressing cariogenic bacterial growth. Together, these findings support the feasibility of small-molecule and functional-group conjugation as a route to engineer multifunctional peptides, with improved proteolytic stability, strong antimicrobial performance, and remineralization potential.

#### 5.3.3 Lipidation modification

To address resistant strains and improve bactericidal efficiency, modification strategies have increasingly shifted toward precise disruption of bacterial membrane architecture. As discussed in Section 3.2, hydrophobicity is a key parameter that determines whether AMPs can effectively anchor to and perturb bacterial membranes. Lipidation, which covalently attaches lipid moieties to specific amino acid residues, provides a more direct means of tuning hydrophobicity than residue substitution, thereby modulating antimicrobial activity (Rounds and Straus, 2020). For relatively hydrophilic peptides, lipidation can increase hydrophobicity to an appropriate extent and promote membrane insertion; in contrast, for highly hydrophobic peptides, additional lipidation may drive excessive aggregation and reduce activity (Lin et al., 2023). C10-KKWW is a lipidated AMP generated by covalently conjugating a C10 fatty acid chain to the N terminus. By increasing peptide hydrophobicity, this modification enhances binding to the phospholipid bilayer and markedly improves targeting efficiency toward the bacterial cell membrane (Xiang et al., 2018). However, some rigid AMPs exhibit insufficient membrane penetration. Yu et al. demonstrated that introducing fatty acid chains is critical for improving the antimicrobial performance of rigid peptides. Their work developed a novel cyclic lipopeptide in which lipidation provides strong hydrophobicity that enables efficient insertion and irreversible disruption of *S. mutans* membranes, leading to leakage of intracellular contents. This strategy not only exploits the membrane-targeting advantage of fatty acid tails but also combines a cyclic scaffold to mitigate the susceptibility of conventional lipopeptides to proteolytic degradation, thereby substantially enhancing antibiofilm activity (Min et al., 2017). For oral translation, lipidation may improve short-contact efficacy and biofilm penetration, but lipid-chain length and conjugation position must be optimized to avoid aggregation, hemolysis, or mucosal toxicity. Its clinical value therefore depends on achieving a favorable balance between membrane anchoring and host compatibility.

### 5.4 Covalent immobilization strategies

Clinical translation of AMPs also requires addressing peptide leaching from restorative materials and the associated deterioration of mechanical properties. Xie et al. proposed a peptide-polymer conjugation (PPC) strategy (Xie et al., 2019, 2020). Rather than relying on simple physical blending, this approach introduces flexible linkers and polymerizable groups such as methacrylates to convert AMPs into resin monomers. Through this chemical modification, peptides are covalently immobilized within the polymer network, which prevents loss of the active component and reduces potential cytotoxicity. At the same time, retained conformational freedom enables effective contact-mediated killing of adherent bacteria, thereby transforming AMPs from freely diffusible agents into functional bioactive components of restorative biomaterials. To enable cross-study comparison, representative optimized anticaries AMPs are summarized in Supplementary Table 1, covering sequences, optimization strategies, mechanisms, models, safety, and stability. This strategy is directly linked to clinical translation because it addresses two practical barriers of AMP-containing restorative materials: uncontrolled peptide release and loss of long-term antibacterial function. It may also improve regulatory definability by fixing the active component within a material interface and clarifying the exposure boundary.

## 6 Drug delivery systems

**Figure 3:**
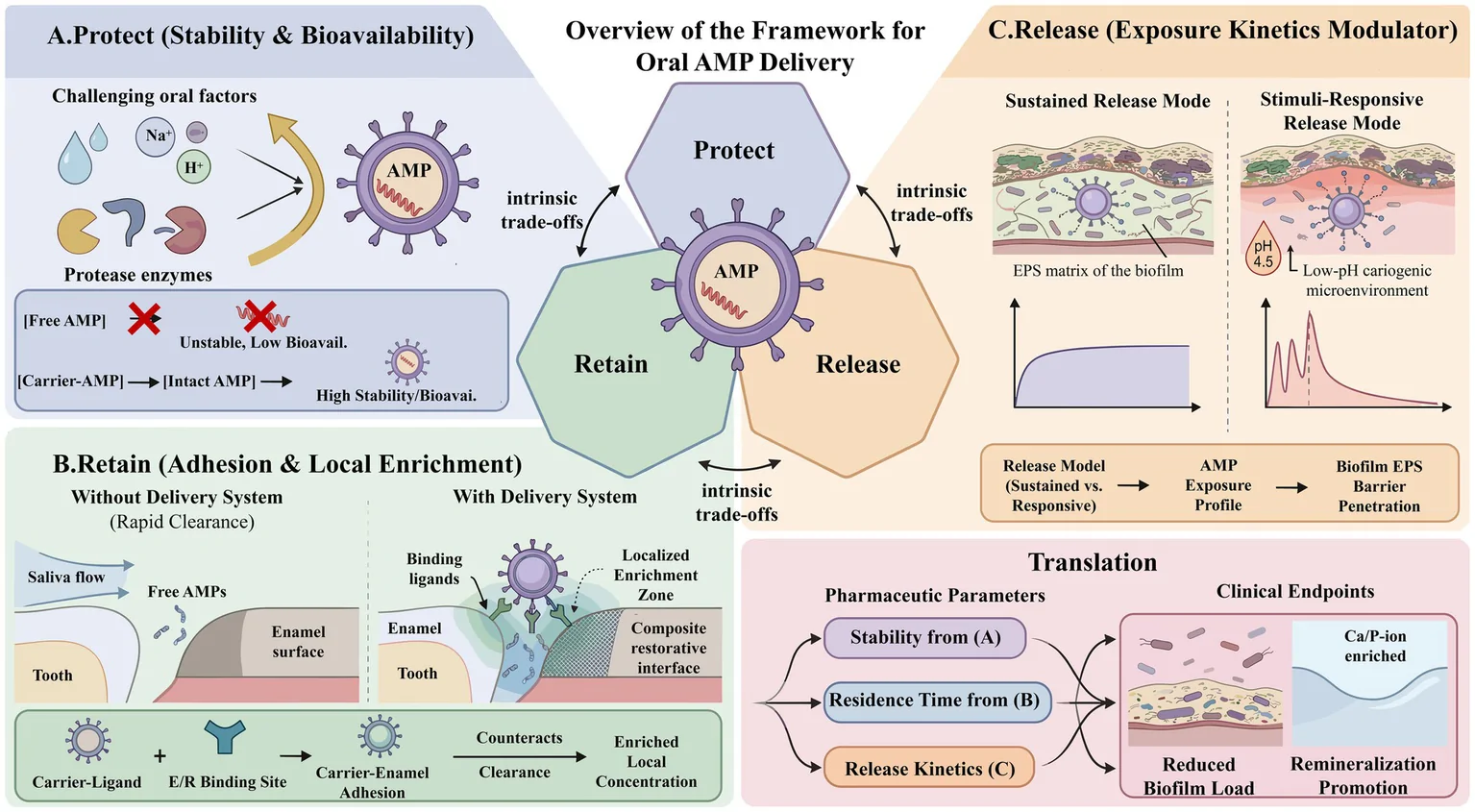
Optimization strategies for antimicrobial peptide delivery in oral applications. **(A)** Protect: improving AMP stability and bioavailability. **(B)** Retain: enhancing local retention and tooth-surface adhesion. **(C)** Release: enabling controlled and sustained AMP release.

Anticaries AMPs face delivery barriers that are highly specific to local oral administration. Continuous salivary clearance markedly shortens effective exposure time, the oral microenvironment (including pH fluctuations and protease activity) promotes inactivation of the payload, and the EPS barrier of mature biofilms restricts drug access to target sites (Deshayes et al., 2022; Thakur et al., 2022). Therefore, the core value of delivery systems should extend beyond drug loading. By enabling retention and localization, protecting peptides from inactivation, and providing triggerable release, delivery platforms can translate *in vitro* peptide activity into sustained therapeutic effects under real oral conditions. As shown in Figure 3, a Protect–Retain–Release framework encapsulates these aims by (i) protecting AMPs from salivary degradation and proteolysis, (ii) retaining and locally enriching AMP at tooth or restorative interfaces, and (iii) enabling sustained or stimuli-responsive release to shape exposure profiles and enhance biofilm penetration.

Simple optimization strategies mainly aim to build a protective barrier through physical encapsulation to improve stability. Zhang et al. reported that liposomal encapsulation of the biomimetic peptide DK5 achieved an encapsulation efficiency of approximately 61.46%. More importantly, after 12 h incubation in human saliva, the liposome group retained 98.77% of the peptide, whereas the free peptide was almost completely degraded. This high stability translated into a marked reduction in enamel mineral loss in an *in vitro* remineralization assay (Zhang Y. et al., 2023). With advances in smart biomaterials, environment-responsive delivery systems have enabled on-demand release within cariogenic microenvironments (Aida et al., 2018). Liu et al. developed a multifunctional nanoparticle (PGE) based on poly(amidoamine)(PAMAM) dendrimers (Liu M. et al., 2025). Under acidic conditions, the particles first release the grafted antimicrobial peptide G(IIKK)4INH2 and the encapsulated EGCG, which, respectively, eliminate bacteria and inhibit matrix metalloproteinase (MMP) activity, thereby protecting the demineralized collagen scaffold. Subsequently, the PAMAM dendrimer acts as a biomimetic template. Through terminal amino groups that adsorb calcium and phosphate ions, it induces *in situ* remineralization within collagen fibrils. This design addresses a key limitation of conventional approaches in which collagen degradation compromises remineralization outcomes.

Among the systems evaluated to date, some platforms provide quantitative data on release and stability, such as liposomal release profiles and salivary stability, or thresholded release and protease protection for pH-sensitive nanoparticles, yet quantitative assessment of tooth-surface retention remains limited (Zhu et al., 2021a; Zhang Y. et al., 2023). Other systems, including immobilized coatings, offer evidence of retention after rinsing but lack systematic release kinetics or long-term exposure profiles (Zhu et al., 2021b). Future studies will be more compelling if three questions are addressed in parallel: (i) how long the formulation remains on tooth surfaces under saliva flow and shear stress; (ii) at what rate the active payload is released or presented during this retention period; and (iii) how these pharmaceutic parameters map onto endpoints such as lesion depth and biofilm outcomes. Overall, delivery systems for anticaries AMPs are evolving from a simplistic goal of increasing local concentration toward a systems-engineering approach that directly targets real-world oral barriers. Representative delivery strategies for anticaries AMPs are summarized in Table 1 for cross-platform comparison.

**Table 1:** Drug delivery systems applied to anticaries AMPs.

Carrier and cargo	Loading/immobilization	Trigger/release feature	Model	References
LCS + D1-23 peptide	Peptide embedded in LCS matrix	Saliva-triggered phase change, slow release	Planktonic culture; *In vitro* monospecies biofilm; Ex vivo tooth	Aida et al. (2018)
Chitosan gel + QP5	Physical entrapment in chitosan hydrogel	pH-responsive ionic association/dissociation	Planktonic culture; *In vitro* monospecies biofilm; Ex vivo tooth	Ren et al. (2019)
PAMAM + surface AMP + EGCG	Covalent G4 amide conjugation on PAMAM	Acid-triggered EG release from cavities	Planktonic culture; *In vitro* monospecies biofilm; Ex vivo tooth; In vivo animal model	Liu et al. (2025a)
Liposomes + DK5	Transmembrane gradient-driven liposomal encapsulation	Sustained release	Ex vivo tooth; In vivo animal model	Zhang et al. (2023d)
PAMAM-C11	Covalent conjugation	No trigger; surface anchoring retention	Ex vivo tooth; In vivo animal model	Cai et al. (2025)
HA/interface + D-GL13K	Electrostatic adsorption self-assembled HA coating	Non-releasing contact-active antibiofilm surface	Planktonic culture; *In vitro* multispecies biofilm; Ex vivo tooth	Moussa and Aparicio (2020)
CNs + HTN3	Ionic gelation entrapment within CNs	Acidic pH swelling-triggered release	Planktonic culture; *In vitro* monospecies biofilm	Zhu et al. (2021a)
CMC nanogel + ClyR + ACP	Electrostatic complexation within CMC nanogel	Low-pH ionization enables sustained release	Planktonic culture; *In vitro* monospecies biofilm; Ex vivo tooth	Zhu et al. (2021b)

## 7 Combination therapy involving anticaries AMPs

The central pathological process of dental caries is the mutual reinforcement between sustained acid production by cariogenic biofilms and localized demineralization. Consequently, antimicrobial suppression alone often fails to produce a durable shift in disease outcomes. Combination regimens involving anticaries AMPs are therefore moving from simply intensifying bactericidal potency toward mechanism-complementary designs. The aim is to concurrently reduce biofilm virulence, limit acidification, and minimize mineral loss, while meeting practical constraints of oral use, including short contact time, salivary clearance, and prolonged exposure at material interfaces. In this context, evidence appraisal for combination strategies should evolve beyond bacterial counts or single metabolic readouts. More informative evaluation should incorporate a continuous chain of endpoints, including acid and extracellular matrix metrics, demineralization outcomes, lesion-related measures, and microbiome-level effects. To facilitate cross-study comparison, we summarize representative combination regimens involving anticaries AMPs in Table 2, highlighting key outcome chains from acid/EPS-related virulence metrics to demineralization and caries lesion endpoints, together with proposed mechanisms, model.

**Table 2:** Mechanism-complementary combination regimens involving anticaries AMPs.

AMP	Combined agent/platform	Key outcomes	Mechanisms	Model	References
Protamine	IPMP (3-methyl-4-isopropylphenol)	↓*S. mutans*; selective killing	Membrane damage by IPMP → protamine intracellular penetration	Planktonic culture	Fujiki and Honda (2020)
GH12 (8 mg/L)	NaF (250 ppm)	↓lactate; delayed pH drop; ↓carbohydrate transport	Membrane disruption → enhanced fluoride intracellular action → PTS suppression	*In vitro* monospecies biofilm	Zeng et al. (2023)
GH12 (8 mg/L)	NaF (250 ppm)	↓caries scores; ↓demineralization; ecological shift	Membrane disruption (GH12) + glycolysis inhibition (fluoride) → ecological shift & EPS suppression	*In vitro* multispecies biofilm; Ex vivo tooth; In vivo animal model	Wang et al. (2022)
GH12	PSeNP + 808 nm laser (photothermal)	↓biofilm CFU	Membrane lysis + localized photothermal heating	Planktonic culture; *In vitro* monospecies biofilm; *In vitro* multispecies biofilm	Jeon et al. (2024)
GA-KR12 peptide	NACP-containing universal adhesive	↓CFU; ↓biofilm biomass; contact-active ↓biofilm; ↑remineralization; ≈bond strength	Membrane-active AMP + Ca/P ion release → antibacterial-remineralization coupling	Planktonic culture; *In vitro* monospecies biofilm; Ex vivo tooth	Mi et al. (2022)
Pep19-2.5	Penicillin/streptomycin (P/S)	↓growth; ↓biofilm; host-safe	Peptide membrane activity + antibiotic bactericidal effect	Planktonic culture; *In vitro* monospecies biofilm	Jannadi et al. (2019)
Pep19-4LF	Penicillin/streptomycin (P/S)	↓growth; ↓biofilm; host-safe	Enhanced membrane interaction → improved antibiotic efficacy	Planktonic culture; *In vitro* monospecies biofilm	Jannadi et al. (2019)
Nisin	Etch-and-rinse adhesive (Single Bond 2)	↓CFU; ↓biofilm activity; ≈bond strength	Lipid II binding → membrane disruption; immobilized contact activity	Planktonic culture; *In vitro* monospecies biofilm; Ex vivo tooth	Su et al. (2018)

↑/↓, increased/decreased vs control; ≈, comparable/no significant difference vs control. CFU, colony-forming units. Control conditions, endpoints, models, and units follow the original studies.

Among existing studies, the combination strategies that most convincingly support anticaries outcomes mainly converge on two lines of evidence. The first line involves mechanism-complementary pairing with cornerstone anticaries agents such as fluoride. Regimens exemplified by GH12 combined with low-concentration fluoride show efficacy in both animal caries lesions and in vitro demineralization endpoints, accompanied by reductions in lactate and insoluble extracellular polysaccharides and a shift in community composition toward a healthier profile. These findings indicate that low-dose combinations may suppress cariogenic biofilms while also improving anticaries outcomes by lowering acid burden and weakening the biofilm matrix (Wang et al., 2022). Furthermore, Zeng et al. (2023) observed in a short-contact biofilm model using 5 min treatment that GH12 plus 250 ppm sodium fluoride produced a greater decrease in lactate and delayed the decline in pH. Transcriptomic analysis and RT-qPCR consistently indicated broad downregulation of carbohydrate transport and metabolic pathways, particularly the PEP-dependent phosphotransferase system. On this basis, it was proposed that GH12-mediated membrane perturbation may enhance intracellular fluoride action, thereby synergistically restricting sugar uptake and metabolic flux. This provides mechanistic support for achieving acid suppression and virulence attenuation with low-dose combinations under short exposure conditions.

The second line emphasizes material-based, interface-resident strategies. Incorporation of AMPs into adhesives can yield predominantly contact-dependent antibacterial effects, but a defined dose window is evident because higher loading can compromise engineering performance, including reduced bond strength, as reported for nisin-modified adhesives (Su et al., 2018). In contrast, composite designs that introduce AMPs together with functional nanocomponents into adhesive systems show stronger multiobjective potential. For example, a universal adhesive containing cecropin-coated Fe3O₄particles inhibited biofilm formation on material surfaces while increasing shear bond strength and was associated with dentin mineral deposition morphology. These results suggest that engineered combinations can, under certain conditions, improve both antibiofilm performance and interfacial properties (Mi et al., 2022).

Overall, combination strategies with greater translational relevance should achieve concurrent reductions in acid production and extracellular matrix formation at low doses and translate these benefits into hard endpoints such as demineralization and caries lesions. At present, the combination of GH12 with low-concentration fluoride provides one of the more complete evidence chains, spanning animal caries outcomes, demineralization-related measures, and phenotypic readouts including lactate and water-insoluble extracellular polysaccharides (Wang et al., 2022). In material-based applications, simply increasing AMP loading may compromise bond strength, whereas composite designs incorporating functional components may enable simultaneous inhibition of biofilms on material surfaces and improvement of interfacial properties. This contrast is exemplified by nisin-modified adhesives versus universal adhesives containing cecropin-coated Fe3O4 particles (Su et al., 2018; Mi et al., 2022). In addition, although transcriptomic data provide mechanistic support for synergistic acid suppression by GH12 combined with low-dose fluoride, current evidence remains constrained by model complexity and the resolution of microbiome profiling, as 16S sequencing often cannot resolve key taxa to the species level. Further validation in systems that more closely recapitulate native dental plaque, coupled with higher-resolution omics, is needed to establish robustness (Wang et al., 2022; Zeng et al., 2023).

## 8 Challenges in clinical translation

### 8.1 Translational evaluation criteria

Clinical translation of anticaries AMPs should not be judged by antimicrobial potency alone, because AMPs commonly face cytotoxicity, susceptibility to protein hydrolysis, and poor pharmacokinetic behavior; delivery systems are therefore needed to improve stability, bioavailability, and controlled release (Zheng et al., 2025). For oral products, translational evaluation should also consider how effectively a formulation can maintain local intraoral exposure: chewing gum increases salivary flow and delivers active agents into the oral cavity (Jiang et al., 2022), whereas plant-cell bioencapsulation may enable slow and sustained local release in chewing-gum or topical formats (Liu et al., 2016).

### 8.2 Manufacturing platforms and production cost drivers

SPPS remains the dominant manufacturing route because it offers precise sequence control, but conventional AMP synthesis has been estimated at US$100–600/g and becomes more difficult as peptide length increases, hydrophobic sequences aggregate, and hazardous solvents and purification steps are required (Chaudhary et al., 2023). Recombinant systems can improve scalability, but short cationic peptides are prone to proteolytic degradation and often require fusion carriers whose removal adds purification steps and may reduce final yield; in comparative bacterial hosts, *E. coli* provided higher yield and higher antimicrobial activity than lactic acid bacteria for HDP-based constructs, whereas lactic acid bacteria remain attractive because they are LPS-free and GRAS (Travé-Asensio et al., 2025). Yeast and plant platforms provide additional options: in Pichia pastoris, optimized expression of peptide K reached 6.67 mg/mL and produced material with activity, stability, and biocompatibility comparable to the synthetic peptide (Zhu et al., 2024), whereas plant systems are considered economical, scalable, and safe (Daniell et al., 2001; Shanmugaraj et al., 2021), and a techno-economic analysis estimated plant-made amidated AMPs at US$74/g, although downstream processing still represented 67% of total cost (Chaudhary et al., 2023). In the oral field, plant-based production is also attractive because current protein-drug costs are strongly driven by expensive fermenters, purification, cold transportation/storage, short shelf life, and sterile delivery requirements, and plant-cell formulations may reduce some of these burdens in topical or chewing-gum formats (Liu et al., 2016).

### 8.3 Quality control, batch consistency, and regulatory definability

Even when production is feasible, translation requires cGMP-compatible manufacturing and batch consistency: commercial peptide APIs are typically supported by three or more validation lots, validated analytical methods, a thoroughly characterized reference standard, impurity limits, and stability and forced-degradation studies, and COA release commonly includes purity, identity testing, endotoxin, bioburden, residual solvents, and mass balance (Pennington et al., 2021). At the pharmaceutical quality-system level, ICH Q10-oriented elements such as Management Review, Change Management System, and Process Performance and Product Quality Monitoring System remain central to maintaining process control and continual improvement (VanDuyse et al., 2021). For peptide products, impurity control is not merely an analytical issue: new peptide-related impurities may increase immunogenicity risk, and regulatory risk assessment should therefore examine whether such impurities introduce T-cell-epitope liability (De Groot et al., 2023). Regulatory definability becomes even more difficult for nanoenabled AMP systems, because AMP-grafted nanocarriers may fall between biopharmaceutical and medical-device classifications, while zeta-potential/particle-size-related safety concerns, non-harmonized nanoparticle standards, lack of international reference materials for potency determination, and the absence of consensus release testing all increase translational uncertainty (Zheng et al., 2025).

### 8.4 Local delivery systems and clinical evidence

Among local delivery systems, GH12 chewing gum showed significant inhibition of *S. mutans* growth and biofilm formation, low cytotoxicity to human gingival epithelial cells, and caries-preventive effects in a rat model; however, the authors emphasized that randomized, controlled, double-blind studies are still needed for clinical application (Jiang et al., 2022). KSL-W chewing gum has advanced further into an FDA-approved randomized Phase 1/2a trial, in which no severe adverse events were observed and plaque and gingival inflammation decreased particularly at doses ≥20 mg, with 30 mg appearing optimal; nevertheless, further research is still required to determine optimal usage and ultimate safety (Ryan et al., 2024). By contrast, GERM CLEAN illustrates the gap between commercial availability and comparative efficacy: its MIC against *S. mutans* was 100% stock solution, the authors considered its antibacterial effect poorer than chlorhexidine (Xiong et al., 2020), and its remineralization effect was inferior to 1,000 ppm NaF, with lower SMHR and greater lesion depth/mineral loss than fluoride treatment (Wang et al., 2023). Taken together, the key translational hurdle is no longer only whether an AMP sequence is active, but whether it can be manufactured reproducibly, defined regulatorily, and supported by comparative local-delivery evidence against established standards of care (Pennington et al., 2021; Wang et al., 2023; Ryan et al., 2024; Zheng et al., 2025).

## 9 Conclusion and future perspectives

Future anticaries AMP design should follow principles centered on interfacial perturbation and ecological modulation. There is no need to indiscriminately increase overall hydrophobicity or peptide length in an attempt to enforce stable transmembrane structures. Instead, design priorities should shift toward optimizing membrane-water interfacial binding and the ability to perturb lipid packing. By integrating stimuli-responsive features and targeting domains, peptides can be engineered for selective suppression of cariogenic bacteria while preserving beneficial commensals and promoting microbiome rebalancing.

However, it should be acknowledged that most current evidence remains preclinical, mainly derived from *in vitro* biofilm models, ex vivo tooth models, simplified multispecies systems, and animal studies. At present, no generalizable clinical framework has been established for AMP-based caries prevention, particularly with respect to standardized exposure regimens, safety endpoints, microbiome-level outcomes, substantivity, and product-format-specific evaluation.

Overall, the application of AMPs in caries prevention and management is at a pivotal transition from empirical trial-and-error toward rational design. With deeper mechanistic understanding of how structure dictates function, future studies should further integrate clinically relevant models, standardized outcome measures, scalable manufacturing, and controlled clinical validation. Such efforts will be essential for developing next-generation smart anticaries therapeutics that can overcome the trade-offs among stability, toxicity, ecological compatibility, and anticaries efficacy.
