# Supplementary material for: Optimization strategies for antimicrobial peptides in dental caries prevention and management: from emerging mechanisms of action to clinical translation
Source: Front Microbiol. 2026 Aug 14;17:1899522. doi: 10.3389/fmicb.2026.1899522 (PMC13522171; doi:10.3389/fmicb.2026.1899522)
Supplement: Supplementary file 1 [file Table_1.docx]

**Supplementary Table 1. Representative optimized anticaries AMPs and related peptide-based systems with reported endpoints, safety, stability, and models.**

| **Peptide** | **Sequence** | **Optimization strategy** | **Primary mechanism/function** | **Antimicrobial endpoint and concentration** | **Safety** | **Stability/tolerability** | **Models** | **References** |
| --- | --- | --- | --- | --- | --- | --- | --- | --- |
| DJK-5@SVA | rivrvriarwqv-SVANAYR | Hybrid peptides;Non-natural amino acid substitution | Direct Antimicrobial Activity;Inhibition of Biofilm Formation and Disruption of Established Biofilms | *S. mutans* MIC100/MBIC100: 31.25 μg/mL; HA/tooth-surface biofilm assays used 20 μM peptide coating and 3-min killing of 3-day biofilms | HGF-1 CCK-8 assay after 24 h exposure to 7.8125–125 μg/mL peptides; no significant viability difference versus control, and 31.25 μg/mL did not compromise cell morphology | HA binding measured by mass depletion after incubation with HA particles (1 mg/mL) at 37 °C and 180 rpm for 3 h; direct saliva/protease stability NR | Planktonic culture;In vitro monospecies biofilm;Ex vivo tooth | (Hu et al., 2024) |
| LR-10 | LRWWLWKLLRRMR-NH2 | Tuning peptide charge;Modulating peptide hydrophobicity;Amphipathicity-tuning strategies | Direct Antimicrobial Activity;Inhibition of Biofilm Formation and Disruption of Established Biofilms | *S. mutans* MIC/MBC: 3.3/6.5 μM; biofilm formation inhibited at 0.8–2× MIC (2.6–6.5 μM); established biofilms treated at 1–10× MIC | Rabbit RBC hemolysis tested at 1.6–52.3 μM for 1 h; low hemolysis at 2× MBC. HGF CCK-8 tested at 0–52.3 μM for 24, 48, and 72 h; no significant viability reduction at 4× MBC | Bactericidal activity retained after 10 s exposure under saliva-relevant NaCl (45–80 mM), acidic pH (5.5–6.0), and clarified saliva conditions; direct retained-percentage stability NR | Planktonic culture;In vitro monospecies biofilm | (Liang et al., 2019) |
| IG-13-1 | IGKLFKRIVQLIK | Length optimization (truncation);Tuning peptide charge;Modulating peptide hydrophobicity;Amphipathicity-tuning strategies | Direct Antimicrobial Activity;Inhibition of Biofilm Formation and Disruption of Established Biofilms | *S. mutans* UA159 MIC: 5.0 µM; MBC: 10.0 µM; MBIC50: 5.91 ± 0.91 µM. | RAW264.7 CC50: 55.11 ± 0.58 µM. | NR. | Planktonic culture;In vitro monospecies biofilm | (Chen et al., 2019) |
| IG-13-2 | IGKLFKRIVQLIL | Length optimization (truncation);Tuning peptide charge;Modulating peptide hydrophobicity;Amphipathicity-tuning strategies | Direct Antimicrobial Activity;Inhibition of Biofilm Formation and Disruption of Established Biofilms | *S. mutans* UA159 MIC: 5.0 µM; MBC: 10.0 µM; MBIC50: 7.58 ± 0.23 µM | RAW264.7 CC50: 52.78 ± 0.25 µM | NR | Planktonic culture;In vitro monospecies biofilm | (Chen et al., 2019) |
| P-113-DPS | AKRHHGYKRKFH-SpSp | Phosphorylation;Tuning peptide charge;Amphipathicity-tuning strategies | Direct Antimicrobial Activity;Inhibition of Biofilm Formation and Disruption of Established Biofilms;Promotion of Remineralization and Prevention of Demineralization | *S. mutans* MIC/MBC: 8/16 µM; mature biofilm killing on tooth slices: 64 µM; anti-adhesion on peptide-coated enamel: 16 µM | MC3T3-E1 CCK-8 after 24 h at 4, 8, 16, and 32 µM | Human saliva incubation 0–4 h; remaining peptide quantified by HPLC | Planktonic culture;In vitro monospecies biofilm;Ex vivo tooth | (Zhou et al., 2021) |
| TVH19 | TKRQQVVGLLWHLLHHLLH-NH2 | Hybrid peptides;Tuning peptide charge;Modulating peptide hydrophobicity;Amphipathicity-tuning strategies | Direct Antimicrobial Activity;Inhibition of Biofilm Formation and Disruption of Established Biofilms;Promotion of Remineralization and Prevention of Demineralization | *S. mutans* MIC/MBC: 64/128 μM; MBIC50: 32 μM; SMIC50 for 1-day-old biofilm viability: 128 μM | HOK cell metabolic activity tested at 1×, 2×, and 4× MBC for 0.5, 1, 2, 4, 6, and 24 h; little effect and no significant difference versus untreated control | TVH19 incubated in human saliva at MBC concentration and 37 °C; HPLC showed peptide remained intact through 3, 6, and 12 h | Planktonic culture;In vitro monospecies biofilm;Ex vivo tooth | (Wang et al., 2019a) |
| DR9/2RR14 (synthetically linked dual hybridized conjugate) | DSSEEKFLRRKFHEKHHSHRGYR | Hybrid peptides;Modulating peptide hydrophobicity;Amphipathicity-tuning strategies | Inhibition of Biofilm Formation and Disruption of Established Biofilms | 96-h *S. mutans* attached-biomass assay on pellicle-coated HA discs; MIC/MBIC/MBEC NR | NR | Enamel immobilization quantified by multiphoton microscopy; coating concentration NR | In vitro monospecies biofilm | (Moussa et al., 2023) |
| DR9-DR9 (duplicated domain) | DSpSpEEKFLRDSpSpEEKFLR | Length optimization (extension);Phosphorylation | Promotion of Remineralization and Prevention of Demineralization | *C. albicans* killing assay: IC50 = 200.6 μM; *S. mutans* IC50 not reached; MIC/MBIC/MBEC NR | NR | Enamel/HA peptide functional-domain coating tested; quantitative retention details NR | Ex vivo tooth;Planktonic culture | (Basiri et al., 2017) |
| DR9-RR14 (hybrid peptide) | DSpSpEEKFLRRKFHEKHHSHRGYR | Hybrid peptides;Phosphorylation | Direct Antimicrobial Activity;Promotion of Remineralization and Prevention of Demineralization | Killing assay: *C. albicans* IC50 = 117.7 μM; *S. mutans* IC50 = 4.7 μM; MIC/MBIC/MBEC NR | NR | Enamel/HA peptide coating tested; quantitative retention details NR | Ex vivo tooth;Planktonic culture | (Basiri et al., 2017) |
| 8DSS-C8-P-113 | DSSDSSDSSDSSDSSDSSDSSDSS-TFFRLF-R-AKRHHGYKRKFH | Hybrid peptides | Direct Antimicrobial Activity;Inhibition of Biofilm Formation and Disruption of Established Biofilms;Interference with Bacterial Metabolism and Stress Adaptation;Promotion of Remineralization and Prevention of Demineralization | Planktonic *S. mutans* killing: 2 μM/mL; anti-adhesion: 8 μM/mL; hydrogel-mediated biofilm eradication and remineralization: 16 μM/mL | MC3T3-E1 cytocompatibility was assessed by CCK-8 after 24 h peptide exposure; all tested 8DSS-C8-P-113 concentrations, including MBC and 16 μM/mL, maintained acceptable cell survival | Human saliva stability: 89.4% intact after 6 h at 37 °C. Flow-water retention: approximately 82.15% retained on tooth surface after 2 h at 30 mL/h. Hydrogel formed at 16 μM/mL | Planktonic culture;In vitro monospecies biofilm | (Zhou et al., 2025) |
| 38a | cyclo(L-Phe-L-Tyr-Bn) | Cyclization;Conjugation with small molecules and functional moieties | Inhibition of Biofilm Formation and Disruption of Established Biofilms | *S. mutans* biofilm formation inhibition: 6–12 μg/mL; *C. albicans* biofilm formation inhibition: 24–48 μg/mL; preformed-biofilm disruption not observed at 96 μg/mL; MIC >192 μg/mL; MBC NR | NR | NR | In vitro monospecies biofilm | (Simon et al., 2019) |
| GHaR | FLQRIIGALGRLF-NH2 | Tuning peptide charge;Amphipathicity-tuning strategies | Direct Antimicrobial Activity;Inhibition of Biofilm Formation and Disruption of Established Biofilms;Interference with Bacterial Metabolism and Stress Adaptation | *S. mutans* MIC/MBC: 1.6/3.1 μM; virulence-factor assays mainly at 1/2× MIC; EPS assay at 1× MIC | HOK cell viability tested up to 100 µM; GHaR showed weak inhibition above 12.5 µM | Physiological-condition activity assessed; stability details NR | Planktonic culture;In vitro monospecies biofilm;In vivo animal model | (Jiang et al., 2023a) |
| GHa11R | FLQHIIGALGRLF-NH2 | Tuning peptide charge;Amphipathicity-tuning strategies | Direct Antimicrobial Activity;Inhibition of Biofilm Formation and Disruption of Established Biofilms;Interference with Bacterial Metabolism and Stress Adaptation | *S. mutans* MIC/MBC: 3.1/6.2 μM; virulence-factor assays mainly at 1/2× MIC; EPS assay at 1× MIC | HOK cell viability tested up to 100 µM; no toxic effect reported for GHa11R | Physiological-condition activity assessed; stability details NR | Planktonic culture;In vitro monospecies biofilm;In vivo animal model | (Jiang et al., 2023a) |
| GHaR6R | FLQRIRGALGRLF | Non-natural amino acid substitution;Tuning peptide charge;Modulating peptide hydrophobicity;Amphipathicity-tuning strategies | Direct Antimicrobial Activity;Inhibition of Biofilm Formation and Disruption of Established Biofilms | *S. mutans* MIC/MBC: 12.5/12.5 µM; killing tested at 1/2×-4× MIC | Hemolysis assay: HL50 increased vs parent; HOEC cytotoxicity tested | NR | Planktonic culture;In vitro monospecies biofilm | (Wei et al., 2020) |
| GHaR8R | FLQRIIGRLGRLF | Non-natural amino acid substitution;Tuning peptide charge;Modulating peptide hydrophobicity;Amphipathicity-tuning strategies | Direct Antimicrobial Activity;Inhibition of Biofilm Formation and Disruption of Established Biofilms | *S. mutans* MIC/MBC: 6.2/6.2 µM; MBIC50: 1.6 µM; MBEC50: 12.5 µM | Hemolysis assay: CSI reported as 27; HOEC cytotoxicity tested | NR | Planktonic culture;In vitro monospecies biofilm | (Wei et al., 2020) |
| GHaR9W | FLQRIIGAWGRLF | Non-natural amino acid substitution;Tuning peptide charge;Modulating peptide hydrophobicity;Amphipathicity-tuning strategies | Direct Antimicrobial Activity;Inhibition of Biofilm Formation and Disruption of Established Biofilms | *S. mutans* MIC/MBC: 6.2/6.2 µM; MBIC50: 3.1 µM; MBEC50: 12.5 µM | Hemolysis assay: HL50 1–5× higher than parent; HOEC cytotoxicity tested | NR | Planktonic culture;In vitro monospecies biofilm | (Wei et al., 2020) |
| GHb3K | FIKHIIGALGHLF-NH2 | Tuning peptide charge;Amphipathicity-tuning strategies | Direct Antimicrobial Activity;Inhibition of Biofilm Formation and Disruption of Established Biofilms;Interference with Bacterial Metabolism and Stress Adaptation | *S. mutans* MIC: 3.1 µM; MBIC: 12.5 µM; EPS assays at 1/2× MIC | HOK CCK-8 at 3.1–100 µM for 2 h; no cytotoxicity up to 100 µM | 30-day serial passage at 1/2× MIC; no MIC increase reported | Planktonic culture;In vitro monospecies biofilm;Ex vivo tooth;In vivo animal model | (Jiang et al., 2024) |
| GH8 | GLLWHLLH-NH2 | Length optimization (truncation);Tuning peptide charge;Modulating peptide hydrophobicity;Amphipathicity-tuning strategies | Direct Antimicrobial Activity | *S. mutans* UA159 MIC/MBC: 256/256 μg/mL; GH8-specific biofilm endpoint NR | Direct mammalian-cell safety NR for GH8 | Direct saliva/protease/surface-retention stability NR | Planktonic culture | (Wang et al., 2017) |
| GH12 | GLLWHLLHHLLH-NH2 | Tuning peptide charge;Modulating peptide hydrophobicity;Amphipathicity-tuning strategies | Direct Antimicrobial Activity;Inhibition of Biofilm Formation and Disruption of Established Biofilms;Interference with Bacterial Metabolism and Stress Adaptation;Enhancement of Commensal Competitive Advantage | MIC: 4.0–8.0 µg/mL; MBC: 8.0–32.0 µg/mL; MBIC50: 8.0 µg/mL; biomass reduction ≥32.0 µg/mL | Primary HGFs exposed to GH12 at 4–128 μg/mL for 5, 60, or 120 min; viability measured after 24 h; only 128 μg/mL for 5 min slightly inhibited proliferation | pH-responsive activity assessed; direct saliva/protease stability NR | Planktonic culture;In vitro monospecies biofilm;Ex vivo tooth | (Wang et al., 2017; Jiang et al., 2021; Li et al., 2021) |
| GH16 | GLLWHLLHHLLHLLHH-NH2 | Length optimization (extension);Tuning peptide charge;Modulating peptide hydrophobicity;Amphipathicity-tuning strategies | Direct Antimicrobial Activity | *S. mutans* UA159 MIC/MBC: >512/>512 μg/mL; subsequent cytotoxicity/biofilm assays NR | NR | NR | Planktonic culture | (Wang et al., 2017) |
| Pm11 | WFKFFKKFFKKWK | Length optimization (truncation);Tuning peptide charge;Modulating peptide hydrophobicity;Amphipathicity-tuning strategies | Direct Antimicrobial Activity | *S. mutans* MIC/MBC: 8/8–16 μg/mL; *S. sanguinis* MIC/MBC: 8/8 μg/mL; *S. sobrinus* MIC/MBC: 8/8 μg/mL; SEM membrane assay used 4 μg/mL for 24 h | Primary HGF CCK-8 assay tested Pm11 at 8 and 32 μg/mL for 5, 30, and 60 min; 8 μg/mL had little effect, while 32 μg/mL inhibited proliferation after 60 min | Direct saliva/protease/surface-retention stability NR | Planktonic culture | (Zhang et al., 2016) |
| GFP-RC101 | - | Cyclization | Direct Antimicrobial Activity | *S. mutans*: MIC 10–20 μg/mL; MBC 80–160 μg/mL. GFP-RC101-specific biofilm working concentration NR | Uptake/localization was imaged in HPDLSC, SCC-1, GMSC, AGK, OBC, and MMSC cells; direct quantitative mammalian-cell viability for GFP-RC101 NR | Plant-made GFP-RC101 was obtained from lyophilized chloroplast material; study-specific saliva/protease stability NR | Planktonic culture | (Liu et al., 2016) |
| DPS-PI | pSpSRRWCFRVCYRGFCYRKCR | Phosphorylation;Conjugation with small molecules and functional moieties | Direct Antimicrobial Activity;Inhibition of Biofilm Formation and Disruption of Established Biofilms | *S. mutans* MIC: 80 μg/mL; MBC NR. Enamel/tooth-slice coating used 2× MIC (160 μg/mL), including 24 h in vitro coating and 1 min rabbit incisor treatment | BMSC proliferation was tested by CCK-8 on enamel slices treated with 2× MIC DPS-PI for 1, 3, and 5 days; no difference versus sterile-water control | Tooth/HA binding and in vivo tooth-retention were tested; quantitative saliva/protease stability NR, and saliva degradation was noted as requiring further study | Planktonic culture;Ex vivo tooth;In vivo animal model | (Zhang et al., 2019) |
| [W7]KR12-KAEK | KRIVQRWKDFLRKAEK-NH2 | Non-natural amino acid substitution;Length optimization (truncation);Tuning peptide charge;Amphipathicity-tuning strategies | Direct Antimicrobial Activity;Inhibition of Biofilm Formation and Disruption of Established Biofilms | MIC: 7.8–31.25 µg/mL; MBC: 15.6–62.5 µg/mL; biofilm CFU reduction tested at 7.8–62.5 and 62.5–500 µg/mL | Direct mammalian-cell safety NR | NR | Planktonic culture;In vitro monospecies biofilm | (Da Silva et al., 2017) |
| CLP-4 | (12-guanidinododecanoyl)-cyclo(Dap-dL-W-dK-dN-dR) | Cyclization;Lipidation;Non-natural amino acid substitution;Tuning peptide charge;Modulating peptide hydrophobicity;Amphipathicity-tuning strategies | Direct Antimicrobial Activity;Inhibition of Biofilm Formation and Disruption of Established Biofilms | *S. mutans* UA159 MIC/MBC: 2.8/6 μg/mL; high-density MIC/MBC: 5/20 μg/mL; MBIC: 5 μg/mL; MBEC: 50 μg/mL | Human RBC hemolysis tested at 8–1000 μg/mL for 1 h; no appreciable hemolysis at MIC, MBC, MBIC, or MBEC. HEPM oral fibroblasts tested at 0–256 μg/mL for 24, 48, and 72 h; effective concentrations showed negligible toxicity | Proteolysis assay with TPCK trypsin or chymotrypsin at 37 °C for 1, 2, 4, 18, and 24 h; 82% and 94% of CLP-4 remained intact after 24 h, respectively | Planktonic culture;In vitro monospecies biofilm | (Min et al., 2017) |
| D-GL13K | Gkiiklkaslkll-NH2 | Non-natural amino acid substitution;Tuning peptide charge;Modulating peptide hydrophobicity;Amphipathicity-tuning strategies | Inhibition of Biofilm Formation and Disruption of Established Biofilms | D-GL13K MIC against multispecies oral bacteria: 1 μg/mL; MBIC/MBEC NR. Dentin-priming/coating assays assessed hydrophobic antimicrobial dentin rather than planktonic *S. mutans* endpoints | CCK-8 after 1 day exposure at 50× MIC showed hDPC viability 88 ± 6% and NIH 3T3 viability 89 ± 6% | D-GL13K release from AAMP-coated dentin was not detected by LC-MS or MALDI-TOF after incubation in artificial saliva at 37 °C under continuous agitation for 24 h and 7 days | Planktonic culture;In vitro multispecies biofilm;Ex vivo tooth | (Moussa et al., 2019b) |
| AVHS@RWWRWW | AVHSRWWRWW | Hybrid peptides | Direct Antimicrobial Activity;Inhibition of Biofilm Formation and Disruption of Established Biofilms | MBIC for *S. mutans* biofilm formation: 40 μg/mL; established-biofilm elimination: 80 μg/mL. Planktonic MIC/MBC NR | Hemolysis assay showed no hemolysis for AVHS@RWWRWW at 0.1536–320 μg/mL; rat H&E assessment reported no systemic-organ toxicity | Direct saliva/protease/surface-retention stability NR | In vitro monospecies biofilm;In vivo animal model | (Yang et al., 2025) |
| LF-1 | WKLLRKAWKLLRKA | Amphipathicity-tuning strategies;Tuning peptide charge;Modulating peptide hydrophobicity | Direct Antimicrobial Activity;Inhibition of Biofilm Formation and Disruption of Established Biofilms | *S. mutans* biofilm MBIC: 8 µM; preformed biofilm killing effective from 8 µM and ~50% at 64 µM | Erythrocyte hemolysis: no hemolytic toxicity, slight increase at 128 µM; 5-week rat topical treatment showed no excess death/weight difference | NR | Planktonic culture;In vitro monospecies biofilm;In vivo animal model | (Luo et al., 2021) |
| LF-2 | GKLIWKLLRKAWKLLRKA | Amphipathicity-tuning strategies;Tuning peptide charge;Modulating peptide hydrophobicity | Direct Antimicrobial Activity;Inhibition of Biofilm Formation and Disruption of Established Biofilms | *S. mutans* biofilm MBIC: 16 µM; preformed biofilm effect from 32 µM and at 64 µM | Erythrocyte hemolysis concentration-dependent; >128 µM caused ~50% hemolysis; 5-week rat topical treatment monitored | NR | Planktonic culture;In vitro monospecies biofilm;In vivo animal model | (Luo et al., 2021) |
| C10-KKWW | C10-KKWW-NH2 | Lipidation | Direct Antimicrobial Activity;Inhibition of Biofilm Formation and Disruption of Established Biofilms;Interference with Bacterial Metabolism and Stress Adaptation | *S. mutans* MIC: 3.9 μg/mL; MBC: 7.8 μg/mL; biofilm treatment: 62.5 μg/mL | Oral fibroblast MTT assay tested 0.9–62.5 μg/mL for 24, 48, and 72 h with negligible viability reduction; rabbit RBC hemolysis was tested across 1.9–125 μg/mL | Direct saliva/protease/surface-retention stability NR | Planktonic culture;In vitro monospecies biofilm | (Xiang et al., 2018) |
| ZXR-2 | FKIGGFIKKLWRSLLA | Length optimization (truncation);Tuning peptide charge;Modulating peptide hydrophobicity;Amphipathicity-tuning strategies | Direct Antimicrobial Activity;Inhibition of Biofilm Formation and Disruption of Established Biofilms | *S. mutans* MIC: 8–16 μM; MBC NR. Biofilm formation was inhibited at 10 μM and 2-day biofilm biomass decreased at 16 μM; 1-day-old biofilms were imaged after 128 μM treatment | Sheep RBC hemolysis tested at 0–32 μM for 3 h; hemolysis was approximately 15% at 8–16 μM and approximately 30% at 4× MIC (32–64 μM) | Direct saliva/protease/surface-retention stability NR | Planktonic culture;In vitro monospecies biofilm | (Chen et al., 2017) |
| GA-KR12 | GA-KRIVQRIKDFLR | Conjugation with small molecules and functional moieties;Tuning peptide charge;Amphipathicity-tuning strategies | Direct Antimicrobial Activity;Inhibition of Biofilm Formation and Disruption of Established Biofilms;Promotion of Remineralization and Prevention of Demineralization | *S. mutans* MIC/MBC: 160/320 μM; enamel treatment used 2× MBC (640 μM). MIC/MBC ranges across tested species were 10–320 μM/20–1280 μM | HGF-1 MTT assay tested GA-KR12 at 80–2560 μM for 24 h; no significant absorbance difference versus negative control | Human-saliva HPLC assay: 82% of GA-KR12 remained after 1 h at 37 °C from an initial 160 μM concentration | In vitro monospecies biofilm;Ex vivo tooth | (Niu et al., 2021c) |
| GI6 | GIDLKI | Length optimization (truncation);Amphipathicity-tuning strategies | Inhibition of Biofilm Formation and Disruption of Established Biofilms | GI6 anti-biofilm working concentration: 50 μg/mL in pH/calcium/glucose/sucrose *S. mutans* biofilm assays; enamel-block model used 1 mg/mL GI6. MIC/MBIC/MBEC NR because GI6 was designed as non-bactericidal anti-biofilm amyloid peptide | Rat safety assessment found no differences in intestinal flora, serum biochemical parameters, or pathological changes of major organs versus control | GI6 was selected as the most stable amyloid hexapeptide and retained anti-biofilm activity across pH 5–9, calcium 1.5–150 mM, and glucose/sucrose-related conditions | In vitro multispecies biofilm;In vitro monospecies biofilm;Ex vivo tooth;In vivo animal model | (Huang et al., 2023) |
| MA-AMPM3 | MA-K_GGG_GLLWHLLHHLLH-CONH2 | Covalent immobilization | Direct Antimicrobial Activity | MA-AMPM3 monomer MIC against *S. mutans*: 7.8 μg/mL; IC50: 4.8 μg/mL. Polymerized MA-AMPM3 discs with 5%, 10%, and 15% cross-linker inhibited *S. mutans*; 20% cross-linker did not | Direct mammalian-cell safety NR | Peptide-polymer conjugates were soaked in water for 5 days before antimicrobial testing and retained activity in the polymerized material; saliva/protease stability NR | Planktonic culture | (Xie et al., 2020) |
| Sp-H5 | phosphoserine-DSHAKRHHGYKRKFHEKHHSHRGY | Phosphorylation;Tuning peptide charge;Amphipathicity-tuning strategies | Direct Antimicrobial Activity;Inhibition of Biofilm Formation and Disruption of Established Biofilms;Promotion of Remineralization and Prevention of Demineralization | *S. mutans* MIC/MBC: 2/4 μmol/mL; biofilm killing from 16× MIC; adhesion inhibition from 2× MIC | HGF-1 MTT assay after 4 h exposure at 2×, 4×, 8×, and 16× MIC showed no significant difference versus control; HGF-1 proliferation on 16× MIC-treated enamel was also assessed for 3 day. | HA binding and Ca/P mineralization assays were performed; peptide-treated enamel slices were incubated overnight at 37 °C. Direct saliva/protease stability NR | Planktonic culture;In vitro monospecies biofilm;Ex vivo tooth | (Zhou et al., 2020) |
| GA-C16G2 | GA-TFFRLF-SFTQALGKGGGKNLRIIRKGIHIIKKY | Conjugation with small molecules and functional moieties;Hybrid peptides;Tuning peptide charge;Amphipathicity-tuning strategies | Direct Antimicrobial Activity;Inhibition of Biofilm Formation and Disruption of Established Biofilms;Promotion of Remineralization and Prevention of Demineralization | *S. mutans* MIC: 3.1 µM; MBC: 20.8 µM | Human gingival fibroblast cytotoxicity evaluated with PrestoBlue assay | Artificial saliva stability measured by HPLC | Planktonic culture;In vitro monospecies biofilm | (Fu et al., 2025) |
| GAPI | GA-RRWCFRVCYRGFCYRKCR | Conjugation with small molecules and functional moieties;Amphipathicity-tuning strategies | Direct Antimicrobial Activity;Inhibition of Biofilm Formation and Disruption of Established Biofilms;Promotion of Remineralization and Prevention of Demineralization | *S. mutans* MIC/MBC: 40/80 μM; L. casei MIC/MBC: 40/160 μM; *C. albicans* MIC/MFC: 20/40 μM; multispecies biofilm/enamel treatment used 160 μM GAPI | HGF-1 CCK-8 assay showed no significant optical-density difference between 640 μM GAPI-treated cells and negative control | Human-saliva HPLC assay: 94.4% of GAPI remained after 60 min incubation at 37 °C | Planktonic culture;In vitro multispecies biofilm;Ex vivo tooth | (Zhang et al., 2023b) |
| G4 | G(IIKK)4I-NH2 | Tuning peptide charge;Amphipathicity-tuning strategies | Direct Antimicrobial Activity;Inhibition of Biofilm Formation and Disruption of Established Biofilms | *S. mutans* MIC: 6 μM; biofilm formation inhibition tested at 16–>32 μM; mature-biofilm biomass reduction ~50% at 8 μM; formal MBIC/MBEC NR | NR | NR | Planktonic culture;In vitro monospecies biofilm | (Zhang et al., 2020) |
| DJK-5 | Ac-(all-D)-VQWRAIRVRVIR-OH | Non-natural amino acid substitution;Tuning peptide charge;Amphipathicity-tuning strategies | Inhibition of Biofilm Formation and Disruption of Established Biofilms;Interference with Bacterial Metabolism and Stress Adaptation | DJK-5 treatment: 40 µL droplet on 1-week multispecies biofilm for 1 min; MIC/MBIC/MBEC NR | NR | Biofilm killing dynamics on restorative materials followed over repeated treatments; stability endpoint NR | In vitro multispecies biofilm | (Hu et al., 2023) |
| GH12-M2 | GLLWHLLHHLLH_GSGGG_K | Covalent immobilization | Direct Antimicrobial Activity | GH12-M2 MIC: 31.3 µg/mL; IC50: 12.75 µg/mL; adhesive discs active at ≥12.5× MIC (390 µg/mL) | Direct mammalian-cell safety NR | Peptide coupling to adhesive and structural folding assessed by CD/FTIR/Raman | Planktonic culture | (Xie et al., 2019) |
| GA-KR12 | GA-KRIVQRIKDFLR | Conjugation with small molecules and functional moieties;Tuning peptide charge;Amphipathicity-tuning strategies | Direct Antimicrobial Activity;Promotion of Remineralization and Prevention of Demineralization | GA-KR12 working concentration: 640 μM, applied for 5 min twice daily during a 7-day *S. mutans* biofilm-remineralization dentine-caries cycle. MIC/MBIC/MBEC NR in this study | Direct mammalian-cell safety NR in this dentine-caries cycling study | Biofilm-remineralization cycling used artificial saliva/remineralization solution at pH 7.0; direct saliva/protease retained-percentage stability NR in this study | Planktonic culture;Ex vivo tooth | (Niu et al., 2021a) |
| Ssp(A4K-A11K) | DYQKKLAAYQKEL | Tuning peptide charge;Amphipathicity-tuning strategies | Inhibition of Biofilm Formation and Disruption of Established Biofilms | Ssp(A4K-A11K) working concentration: 650 μM for anti-adhesion and biofilm inhibition on saliva-coated polystyrene plates and hydroxyapatite disks; 1300 μM showed comparable adhesion inhibition. MIC/MBC/MBIC/MBEC NR | Direct mammalian-cell safety NR. At 650 μM, Ssp(A4K-A11K) did not affect planktonic *S. mutans* growth and had limited effects on S. mitis and S. salivarius adherence/biofilm formation | Peptide binding to salivary gp340/SRCRP2 was tested; anti-biofilm activity persisted through 14 h in early biofilm assays. Direct saliva/protease degradation data NR | In vitro monospecies biofilm | (Ito et al., 2017) |
| LH12 | GLLHLLHHLLHH-NH2 | Tuning peptide charge;Modulating peptide hydrophobicity;Amphipathicity-tuning strategies | Direct Antimicrobial Activity;Inhibition of Biofilm Formation and Disruption of Established Biofilms;Enhancement of Commensal Competitive Advantage | Against cariogenic bacteria, LH12 MIC: 8–16 μg/mL and MBC: 16–32 μg/mL. *S. mutans* biofilm mass was reduced by 84.04% at 8 μg/mL, and biofilm formation was fully inhibited at 16–32 μg/mL; 32 μg/mL reduced viable *S. mutans* in preformed biofilms | HGEC CCK-8 after 45 min showed only slight cytotoxicity at 128 μg/mL (9.64% inhibition, no significant OD450 reduction). Hemolysis was absent below 64 μg/mL and 17.15% at 128 μg/mL | pH-responsive activity was tested in phosphate/citrate buffer; antimicrobial activity was stronger at pH 5.5 than pH 7.2. Direct saliva/protease stability NR | Planktonic culture;In vitro multispecies biofilm;Ex vivo tooth | (Jiang et al., 2023b) |

**Footnotes.** MIC, minimum inhibitory concentration; MBC, minimum bactericidal concentration; MBIC, minimum biofilm inhibitory concentration; MBEC, minimum biofilm eradication concentration; EPS, extracellular polysaccharides; PTS, phosphotransferase system; HA, hydroxyapatite; NR, not reported.
